# Identification of over- and undertreatment in the Dutch national cervical cancer screening program: A data linkage study at the hospital level

**DOI:** 10.1016/j.pmedr.2023.102134

**Published:** 2023-02-10

**Authors:** Maarten D. Vink, Geeske Hofstra, Xander Koolman, Ruud L. Bekkers, Albert G. Siebers, Folkert J. van Kemenade, Koen B. Böcker, Michiel ten Hove, Eric J. van der Hijden

**Affiliations:** a Department of Health Economics, School of Business and Economics & Talma Institute, Vrije Universiteit, Amsterdam, the Netherlands; b Department of Obstetrics and Gynecology, Isala, Zwolle, the Netherlands; c Department of Obstetrics and Gynecology, Catherina Cancer Institute, Catharina Hospital, Eindhoven, the Netherlands; d Department of Obstetrics and Gynecology, GROW School for Oncology and Developmental Biology, Maastricht University Medical Center, Maastricht, the Netherlands; e PALGA, Houten, 3991SZ, the Netherlands; f Department of Pathology, Erasmus MC, University Medical Center Rotterdam, Rotterdam, 3000 CA, the Netherlands; g National Health Care Institute, Diemen, 1112 ZA, the Netherlands; h Vektis, Zeist, 3708 JE, the Netherlands; i Zilveren Kruis Health Insurance, Leusden, the Netherlands

**Keywords:** hrHPV, high-risk human papillomavirus, CIN, cervical intraepithelial neoplasia, LEEP, loop electrosurgical excision procedure, Cervical intraepithelial neoplasia, Quality indicators, Guideline adherence, Cervical cancer screening, National screening program, Health policy, Physician practice patterns

## Abstract

Research into the quality of cancer screening programs often lacks the perspective of clinicians, missing insights into the performance of individual hospitals. This retrospective cohort study aimed to identify guideline deviation (specifically, overtreatment and undertreatment) related to the cervical cancer screening program in Dutch hospitals by deterministically linking nationwide insurance data with pathology data for cervical intraepithelial neoplasia (CIN). We then constructed quality indicators using the Dutch CIN guideline and National Health Care Institute recommendations to assess compliance with CIN management, treatment outcomes, and follow-up, using an empirical Bayes shrinkage model to correct for case-mix variation and hospitals with few observations. Data were linked for 115,899 of 125,751 (92%) eligible women. Overtreatment was observed in the see-and-treat approach (immediate treatment) for women with low-grade referral cytology (4%; hospital range, 0%–25%), CIN ≤ 1 treatment specimens (26%; hospital range, 10%–55%), and follow-up cervix cytology ≥2 months before the guideline recommendation after treatment for CIN 2 (2%; hospital range, 0%–9%) or CIN 3 (5%; hospital range, 0%–19%). By contrast, undertreatment was observed for treatment within 3 months after a CIN 3 biopsy result (90%; hospital range 59%–100%) and follow-up ≥2 months beyond the guideline recommendation after treatments for CIN 2 (21%, hospital range 7%–48%) and CIN 3 (20%, hospital range 7%–90%). In conclusion, we found evidence of CIN overtreatment and undertreatment in all measured domains at the hospital level. Guideline adherence could be improved by implementing the developed indicators in an audit and feedback instrument for use by healthcare professionals in routine practice.

## 1 Introduction

Cervical cancer is common and causes significant mortality and morbidity worldwide (Pimple and Mishra, 2022). Invasive disease develops over multiple steps, with high-risk human papillomavirus (hrHPV) a recognized cause of pre-malignant cervical intraepithelial neoplasia (CIN) (Walboomers et al., 1999). Many countries have implemented national screening programs to identify CIN before it can evolve to cervical cancer.

In 2017, the Netherlands changed its cytology-based screening program to an hrHPV-based program that refers women with hrHPV-positive cervical cytology showing atypical squamous cells of undetermined significance or worse for colposcopy (Loopik et al., 2020). CIN lesions found during colposcopy might warrant a loop electrosurgical excision procedure (LEEP) (Federatie Medisch Specialisten, 2021). Despite clear recommendations for the diagnosis and treatment of CIN, the Dutch National Health Care Institute published a report in 2019 that detailed variation among regions and laboratories in the medical care these women receive (Zorginstituut Nederland, 2019). Notably, 15% of women with low-grade abnormal referral cytology received immediate treatment despite a two-step strategy being recommended (i.e., take a cervical biopsy and use the result to decide on treatment). Treatment deviations were identified in 8% of women with a CIN 1 biopsy who received unnecessary treatment and 13% with a CIN 3 biopsy who received no treatment. During follow-up, the frequency and timing of cervical cytology also deviated from the guideline (Zorginstituut Nederland, 2019). However, this report only gave feedback at the regional level, leaving uncertainty about whether or how healthcare professionals should adapt their clinical practice in each hospital. Practice variation can certainly be warranted when it results from differences in patient characteristics or preferences (Wennberg, 2002). However, when hospitals differ significantly in their adoption of clinical guidelines, healthcare professionals should critically assess whether care can be improved. Understanding when these treatment deviations occur is an important first step to differentiating appropriate from inappropriate deviation, and may even show where current guidelines need to be adjusted. From a societal perspective, unwarranted practice variation may also increase healthcare costs and decrease healthcare access (Wennberg, 2002, Duell et al., 2018). Specifically, overtreating CIN could lead to avoidable adverse obstetric outcomes (e.g., pre-term birth and the associated short- and long-term complications) (Arbyn et al., 2008, Kyrgiou et al., 2016, Loopik et al., 2021, Vogel et al., 2018), whereas undertreatment could lead to avoidable cervical cancer morbidity and mortality (McCredie et al., 2008).

Researchers in other countries have shown the utility of national database linkage between health insurance claims data and cancer registries when analyzing practice variation in cervical cancer screening (Bui et al., 2021, Watanabe et al., 2018). A similar approach may help improve the management of CIN among Dutch hospitals. The present research therefore aimed to identify CIN guideline deviation (e.g., overtreatment and undertreatment) at the hospital level, using specifically developed quality indicators to provide healthcare professionals with information about their clinical performance.

## 2 Materials and methods

### 2.1 Study design

We conducted a retrospective, cross-sectional, data linkage study in the Netherlands that included all women referred to the gynecologist for abnormal cervix cytology between January 1st, 2018 and December 31st, 2019. Women were identified by Diagnosis Treatment Combination code G19 (cervical pathology) and were included irrespective of the indication for the abnormal cervix cytology. This could either be through the national screening program or due to specific complaints (e.g., postcoital or intermenstrual bleeding).

Data linkage was between the Dutch nationwide databases of Vektis and PALGA. Vektis is the executive agency of *Zorgverzekeraars Nederland*, the umbrella organization of all health insurance companies in the Netherlands. Its database provides information on healthcare costs related to the Dutch Health Insurance Act (Vektis, 2021), including the treating hospital and the medical procedures performed, with the most recent data available to December 31st, 2019. By contrast, PALGA (the Dutch Pathological Anatomical National Automated Archive) could provide the cytological and histological results for each woman linked to the Vektis database from January 1st, 2018, to September 25th, 2020 (PALGA, 2021). We used the longer time-frame offered by PALGA to collect follow-up data for patients with treatment data in Vektis to December 31st; 2019.

The study was approved by the scientific committee and privacy committees of PALGA and Vektis. The study was exempt from institutional review board approval and the need for written patient consent because data were gathered retrospectively and analyzed anonymously. However, to reduce the risk of identifying individual women, we could not publish data for indicators that involved <20 women.

### 2.2 Outcomes

We derived three categories of 12 quality indicators from Dutch CIN guideline recommendations (Federatie Medisch Specialisten, 2021) and National Health Care Institute indicators (Zorginstituut Nederland, 2021). Table A1 shows the definitions of all indicators with the corresponding advice from the Dutch guideline, and Appendix A shows the data definitions. The first category (indicators 1–5) encompasses CIN management, describing whether the clinician treated correctly based on the available cytological or histological information. The second category (indicators 6–8) reflects CIN treatment results, such as the treatment specimen or percentage of women with a normal or class 2 (atypical) Pap smear on follow-up cervical cytology after treatment for CIN 2 or 3 within 8 months of treatment (follow-up advised 6 months after treatment, with a 2 month margin) as a proxy of the quality of the LEEP. The third category (indicators 9–12) indicates the timeframe for cervical cytology follow-up after treatment or colposcopy with expectant management (this can be within a margin of 2 months around the guideline recommendation). Because of the expected low prevalence of adenocarcinoma in situ, this histological result was labeled CIN 3.

For the indicators on the see-and-treat high-grade referral cytology (indicator 2) and treatment decision after CIN 2 biopsy (indicator 4), we performed sub-analyses by age group (<40 years and ≥40 years) as a proxy for having completed childbearing. We opted to perform sub-analyses here, rather than altering the definition of indicators, because the guideline advises more restraint when offering treatments to women who might want to become pregnant in the future.

### 2.3 Data linkage and security

Appendix B shows all steps in the deterministic linkage of PALGA and Vektis datasets that used pre-defined rules to classify whether records from different datasets referred to the same woman (Harron et al., 2020). Before receiving data from the two databases, Zorg TTP (Zorg TTP, 2021) generated pseudonyms based on four-digit postal codes and dates of birth for blind linkage. Vektis removed cases with similar dates of birth and postal codes before transferring the data to Zorg TTP, minimizing the risk of an incorrect match. PALGA selected women with cervical cytology or histology results registered during the study period, providing Zorg TTP with a list of their dates of birth and four-digit postal codes. Zorg TTP then sent PALGA a list of pseudonyms for women with a match in the Vektis dataset, and PALGA provided the necessary pathology data. Finally, Zorg TTP removed or pseudonymized any privacy-sensitive information before transferring the two datasets to our research group. The research team checked each dataset for inconsistencies before data linkage to minimize the risk of incorrect matches. If a woman visited multiple hospitals during the study, we allocated each hospital to each observation (colposcopy, LEEP or cold-knife conization) and excluded cases a specific hospital could not be assigned. To limit registration bias, we only used data for a specific indicator if information on the intervention corresponded between the datasets.

Data security was guaranteed with a privacy sending module for all data transfers between PALGA, Vektis, Zorg TTP, and our research group. The participating university required data analysis on an offline computer with storage in a secure offline archive after completion. Although we could only publish anonymous information per hospital, individual healthcare professionals could gain preliminary insights using the audit results and feedback for their own hospital. To ensure data security and privacy, pseudonymized audit and feedback information reports were generated and published on the secure website of the Dutch Society of Obstetricians and Gynaecologists. Hospitals could request Zorg TTP access codes to gain insights about their own report. National outcome data were not published.

### 2.4 Statistical analysis

SAS (version 9.4; SAS Institute Inc., Cary, NC, USA) was used for data storage and analysis. We calculated the hospital-specific crude rate for each indicator and the case-mix adjusted rate (AR) for indicators of the CIN management strategy, and we present risk estimates as average, minimum, and maximum rates. Funnel plots were constructed using SAS to examine the variation between Dutch institutions for each of the 12 indicators, maximizing reliability by excluding institutions that treated <500 women with CIN between 2018 and 2019. To account for differences in observations between hospitals, we used a Bayes shrinkage model for management indicators because a treatment decision for a few women could significantly affect the outcomes of indicators for hospitals with relatively few cases (i.e. slightly more than 20 cases). We estimated these models using random effects logistic regression, drawing the hospital effects from a (random) normal distribution. Outcomes were estimated with and without adjustment for the covariates age and hrHPV status, allowing case-mix correction for indicators of the CIN management strategy. The hospital predicted value was based solely on an intercept and a hospital random effect. This intercept was chosen to ensure that the mean of the predicted means per hospital was mean preserving.

Sensitivity analyses were performed to compare the groups included and excluded during data linkage. Data for both groups were only available in the Vektis dataset, allowing comparison by age, colposcopies, and treatments. Furthermore, analyses on see-and-treat low grade referral cytology (indicator 1), treatment decision after biopsy CIN 1 (indicator 3) and treatment specimen (indicator 6) were stratified by age group <40 years and ≥40 years.

## 3 Results

### 3.1 Data linkage

We could deterministically link 92% (115,899 of 125,751) of the women seen by a gynecologist with abnormal cervical cytology during the study period. Appendix B presents the number excluded per step in the data linkage procedure, which resulted in the further exclusion of 3,019 women treated in 23 hospitals serving <500 women during our study period (final dataset = 112,880 women). At the hospital level, 4%–10% of women could not be linked, though for one outlier, linkage was not possible for 31% of women. Concerning the risk of registration bias, exclusions due to intervention type (LEEP or biopsy) did not correspond between the datasets involving 13% (hospital range, 0%–67%) of the observations in the Vektis dataset and 15% (hospital range, 4%–66%) in the PALGA dataset. Table 1 shows the baseline characteristics of the study participants and hospitals. Sixty (83%) unique hospitals requested preliminary results for their own hospital, to be used as audit and feedback within their own hospital.

**Table 1:** Baseline characteristics of study population.

n (%)	112,880
Age, y, median (range)	39 (14–97)

Hr-HPV status, n (%)	
- Negative	30,050 (27 %)
- Positive	71,813 (64 %)
- Unknown / invalid	11,017 (10 %)

Referral cytology, n (%)	
- Low-grade	32,913 (63 %)
- High-grade	19,408 (37 %)

Histologic results from biopsy, n (%)	
- CIN ≤ 1	27,185 (57 %)
- CIN 2	10,837 (23 %)
- CIN 3	7,292 (15 %)

Final histological results from treatment specimen, n (%)	
- CIN ≤ 1	5,399 (26 %)
- CIN 2	5,637 (27 %)
- CIN ≥ 3	9,520 (46 %)
- Other	61 (0 %)
Number of hospitals	72
Women per hospital, n (median; range)	113,886 (1349; 505–4,510)
Colposcopies per hospitals, n (median; range)	47,449 (536; 47–3,112)
LEEPs per hospital, n (median; range)	20,110 (242; 7–824)
Cold knife conization per hospital, n (median; range)	507 (7; 0–47)

### 3.2 Management strategy

Appendix C provides the funnel plots for all indicators and Appendix D shows the empirical Bayes shrinkage models for indicators 1–5 and the crude results of all indicators per hospital, including the sub-analyses by age group.

**Fig. 1:**
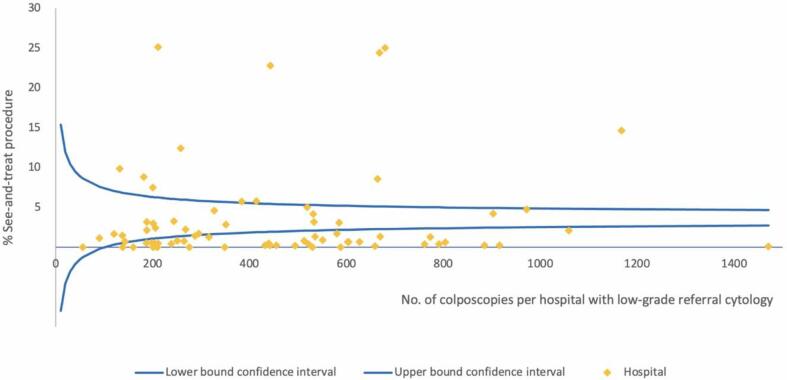
Funnel plot see-and-treat approach for low-grade referral cytology.

**Fig. 2:**
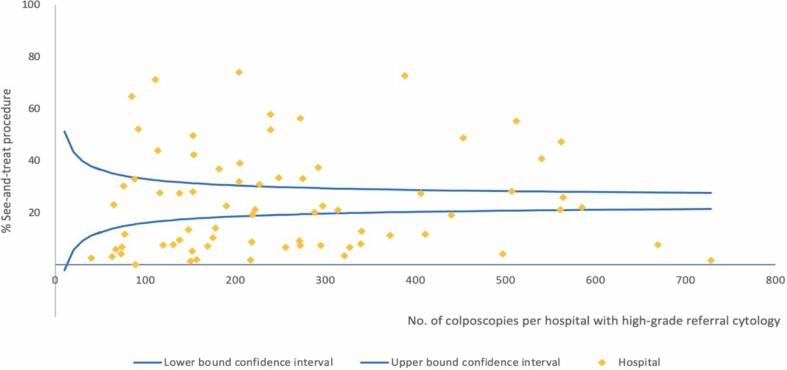
Funnel plot see-and-treat approach for high-grade referral cytology.

Wide variation existed in the management strategies of women with low- and high-grade referral cytology. Fig. 1, Fig. 2 show the funnel plots for these indicators. The see-and-treat approach (i.e., excision within 3 months after referral cytology without prior biopsy) was executed in 4% of women with low-grade referral cytology (hospital range, 0%–25%; N=32,457; AR, 0%–27%) and in 25% of women with high-grade referral cytology (hospital range, 0%–74%; N=18,514; AR, 1%–75%; <40 years, 19%; ≥40 years, 33%). When following the two-step procedure with excision within 3 months after biopsy, 3% underwent a LEEP when cervical biopsy showed CIN ≤ 1 (hospital range, 0%–11%; AR, 1%–10%), 41% when biopsy showed CIN 2 (hospital range, 4%–78%; AR, 11%–79%; <40 years, 28%; ≥40 years, 70%), and 90% when biopsy showed CIN 3 (hospital range, 59%–100%; AR, 62%–96%).

### 3.3 Treatment outcomes

**Fig. 3:**
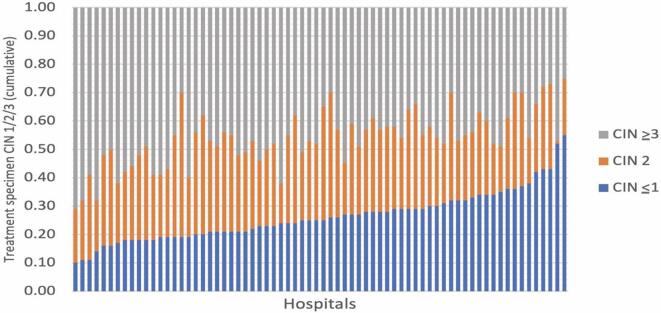
Contribution of treatment specimen CIN ≤1, 2, and ≥3 per hospital, ranked by CIN 1.

Of the 112,880 included women, 19,873 (18%) received 20,617 treatments according to both datasets. Of these, 20,110 (98%) and 507 (2%) received LEEP and cold knife conization, respectively. The contribution of cold knife conization to CIN treatment (including adenocarcinoma in situ) was 0%–15% per hospital. Fig. 3 shows the contribution of CIN grade to treatment specimens for each hospital. After treatment, specimens showed CIN ≥3, 2, and ≤1 in 46% (hospital range, 25%–71%), 27% (hospital range, 1%–51%), and 26% (hospital range, 10%–55%), respectively. Among the 9,461 women who underwent two-step procedures, 62% of treatment specimens agreed with the biopsy result. Appendix D shows the effects of indicators on the normalization rate (indicator 7–8), defined as the first follow-up cervical cytology showing class ≤2 (normal or atypical) on Pap smear follow-up after CIN treatment. Table A2 shows the histological outcome for each excisional procedure by the biopsy result.

### 3.4 Follow-up

In total, 29,314 women underwent colposcopy with or without a biopsy showing CIN 1 histology and received no treatment. During follow-up, 35% (hospital range, 4%–65%) underwent cervical cytology testing within the correct timeframe of 10–14 months, while 38% (hospital range, 9%–88%) did so within 10 months and 27% (hospital range, 8%–68%) did so after 14 months. Another 4,175 women received no treatment after colposcopy for a CIN 2 biopsy result, with 15% (hospital range, 0%–46%) having follow-up within the correct timeframe of 10–14 months, 65% (hospital range, 24%–93%) doing so within 10 months, and 21% (hospital range, 5%–48%) doing so after 14 months. Of the 5,637 women treated for CIN 2, 77% (hospital range, 48%–90%) had follow-up cervical cytology within the correct timeframe of 4–8 months, while 2% (hospital range, 0%–9%) and 21% (hospital range, 7%–48%) had follow-up within 4 months and after 8 months, respectively. Finally, of the 9,328 women treated for CIN 3, 75% (hospital range, 10%–93%) had follow-up within the correct timeframe of 4–8 months, while 5% (hospital range, 0%–19%) had follow-up before 4 months and 20% (hospital range, 7%–90%) had follow-up after 8 months.

### 3.5 Sensitivity analyses

The Vektis and PALGA datasets could not link 9,852 (8%) women. Although the linked and unlinked groups did not differ by age (39 vs 40 years), they did differ by colposcopies (0.51 vs 0.70) and LEEPs (0.16 vs 0.23) performed per person, with fewer procedures when datasets could be linked. Appendix D shows outcomes of indicator 2,3 and 6 stratified by age.

## 4 Discussion

Despite a long tradition of guideline implementation in the Netherlands, our results indicate areas of deviation for the management of CIN across 12 quality indicators covering CIN management, treatment outcomes, and follow-up. After correction for hospital case mix, our data reveal areas where the care quality can be improved. Most hospitals treated women with low-grade referral cytology by the two-step approach, performing colposcopy and biopsy before treatment (97%), but we also identified several hospitals (6%) that used a see-and-treat approach for more than 20% of women. Although the see-and-treat approach is known to produce overtreatment (Loopik et al., 2020), we found that women who received the two-step approach also experienced overtreatment. The percentage aged <40 years (a proxy for potential future pregnancy) who received treatment for a CIN 2 biopsy result varied from 3% to 76% by hospital. Guideline deviation also existed during surveillance, with evidence that cervical cytology was often taken too early after colposcopy or LEEP. International initiatives, such as the Choosing Wisely campaign, have raised awareness about the overuse of colposcopies, biopsies, and treatments in cervical cancer screening programs that are associated with increased healthcare costs and disease-specific distress for women (The American College of Obstetricians and Gynecologists, 2016). However, we also observed undertreatment. Although the Dutch guideline recommends treatment for CIN 3, four hospitals performed LEEP in <80% of women with this biopsy result. This may be explained by participation in a Dutch trial assessing imiquimod for the treatment of CIN 3 (Hendriks et al., 2022), performing surgical treatment after 3 months, and registration bias. When imiquimod becomes a more common treatment for CIN, quality indicators should be adapted to accommodate its use. Given that Vektis has access to pharmacy claims data, this would require minimal input.

Physician and patient factors each have the potential to cause treatment variation. On the one hand, some physicians may perform more treatments due to perceived pressure to accommodate patients, while others might have strong beliefs about the efficacy of a particular therapy. Differences in malpractice concerns or financial considerations could explain the variations between physicians (Cutler et al., 2019). On the other hand, patients’ attitudes and beliefs can significantly affect treatment decisions, emphasizing the need for better patient education (Edmonds, 2014). In a randomized experiment, Dodd et al. showed that 79% of women of childbearing age with a CIN 2 result preferred active surveillance to immediate treatment after counseling (Dodd et al., 2021). We believe that effective counseling could produce similar outcomes in the Netherlands.

Audit cycles with healthcare professional feedback and input can reduce treatment variation by providing key information about clinical performance over a specific period (Ivers et al., 2014). Literature shows that audit and feedback are most effective when the recipient has control over the clinical performance goals, especially when that feedback is provided sequentially in an audit cycle (Brown et al., 2019). However, data recruitment needs to be efficient if hospitals are to receive regular and up-to-date information on their clinical performance. Routinely collected data represent a viable source of such information because they require no additional time investment, with the potential to increase their value by linkage to other relevant data sources. Our study shows that quality indicators derived in this way can provide insights into healthcare quality in the Netherlands. During the study, more than 80% of the hospitals requested information on their own preliminary outcomes, suggesting that our quality indicators were considered a valuable audit and feedback instrument.

A desirable quality indicator should not produce a black or white outcome (Mercuri and Gafni, 2017). Indeed, a practicing clinician might validly deviate from a guideline for an individual patient, even where the guideline explicitly recommends performing or withholding treatments for specific indications. Quality indicators might also fail to capture all the information of interest. Consequently, literature on audit and feedback advocates setting a specific goal per quality indicator to account for the fact that compliance scores of 0% and 100% are neither desirable nor achievable in all instances (Brown et al., 2019). Our 12 indicators cover the entire journey of women who are referred to gynecologists for CIN, providing an overview of the diagnostic, treatment, and follow-up process. This approach has the potential to increase the value of audit and feedback at the hospital level by allowing the evaluation of indicators in context.

Our study has several strengths. First, using two nationwide databases and an effective deterministic linkage algorithm allowed us to provide a nearly complete dataset representative of the general population. PALGA could also provide data for all registered cytological and histological results, irrespective of the source, ensuring a complete dataset even if women switched between hospitals or the general practitioner took follow-up samples. Women could also change their health insurance company without affecting the dataset because Vektis covers all companies in the Netherlands. Second, our ability to track most referred women meant that the results had minimal insensitivity to sampling variability and chance findings. Although some hospitals produced few observations, we excluded those with <500 women and used empirical Bayes shrinkage models to correct for low numbers in each indicator category, thereby preventing chance findings from affecting the outcomes.

An important limitation of our study is that we could not use social security numbers due to privacy concerns, meaning that we could not link 8% of the women in the Vektis dataset to the PALGA dataset. Although sensitivity analysis showed that the groups of excluded and included women did not differ by age, women in the excluded group received fewer treatments per person. This may be explained by a comparatively higher number of women undergoing treatment before the study period or by migration to another postal code. When that occurred, a return to their initial hospital with a follow-up CIN result could not be linked back if the woman did not inform the hospital of their updated postal code. Nevertheless, we believe these women received similar treatment and that their exclusion did not affect the representativeness of our outcomes. Finally, a new Dutch clinical guideline on CIN was published in September 2021. This introduced several changes that will necessitate minor adaptations to the quality indicators used in the present study, such as the integration of hrHPV status in the follow-up of CIN. Future studies should seek to align our quality indicators with the new guideline.

## 5 Conclusions

Women with CIN do not receive consistent treatment among Dutch hospitals, with examples of undertreatment and overtreatment evident throughout their management and follow-up. Linking data from routinely collected sources may facilitate audit and feedback loops that can improve the quality, efficiency, and equity of healthcare provision. Future research should analyze whether implementing audit and feedback in daily clinical practice will result in improved guideline adherence and care quality for women with CIN.

## Authors contributions

MV and RB designed the study. AS, MH, FK, EH, and MV were involved in the compliance procedure to retrieve the data from PALGA and Vektis. GH analyzed the data and XK provided feedback to the statistical analyses performed. MV and GH drafted the manuscript. XK, EH, AS, KB, RB, and FK revised the manuscript. All authors reviewed and approved the final manuscript.

## Funding

This work was funded by Health Evaluation and Appropriate Use (no specific grant ID). The funder website URL is: https://zorgevaluatiegepastgebruik.nl. The funder had no role in the study design, data collection and analysis, decision to publish, or preparation of the manuscript.

## Declaration of Competing Interest

The authors declare that they have no known competing financial interests or personal relationships that could have appeared to influence the work reported in this paper.

## Data Availability

The authors do not have permission to share data.

## Supplementary data

Supplementary Appendix A Data definitions

Supplementary Appendix B Data linkage procedure

Supplementary Appendix C Funnel plots

Supplementary Appendix D Outcome per hospital

## References

[b0005] Arbyn M., Kyrgiou M., Simoens C., Raifu A.O., Koliopoulos G., Martin-Hirsch P., Prendiville W., Paraskevaidis E. (2008). Perinatal mortality and other severe adverse pregnancy outcomes associated with treatment of cervical intraepithelial neoplasia: meta-analysis. Br. Med. J..

[b0010] Brown B., Gude W.T., Blakeman T., van der Veer S.N., Ivers N., Francis J.J., Lorencatto F., Presseau J., Peek N., Daker-White G. (2019). Clinical Performance Feedback Intervention Theory (CP-FIT): a new theory for designing, implementing, and evaluating feedback in health care based on a systematic review and meta-synthesis of qualitative research. Implement. Sci..

[b0015] Bui C., Choi E., Suh M., Jun J.K., Jung K.W., Lim M.C., Choi K.S. (2021). Trend analysis of process quality indicators for the Korean National Cervical Cancer Screening Program from 2005 to 2013. J. Gynecol. Oncol..

[b0020] Cutler D., Skinner J.S., Stern A.D., Wennberg D. (2019). Physician beliefs and patient preferences: A new look at regional variation in health care spending. Am. Econ. J.: Econ. Policy.

[b0025] Dodd R., Cveijc E., Bell K., Black K., Bateson D., Smith M.A., Mac O.A., McCaffery K.J. (2021). Active surveillance as a management option for cervical intraepithelial neoplasia 2: An online experimental study. Gynecol. Oncol..

[b0030] Duell D., Lindeboom M., Koolman X., Portrait F. (2018). Practice variation in long-term care access and use: The role of the ability to pay. Health Econ..

[b0035] Edmonds B.T. (2014). Shared decision-making and decision support: their role in obstetrics and gynecology. Healthcare Manage. Strategies.

[b0040] Federatie Medisch Specialisten. De richtlijn CIN, AIS en VAIN (Cervicale intra-epitheliale neoplasie (CIN), adenocarcinoom in situ (AIS) en vaginale intra-epitheliale neoplasie (VAIN)). 2021.

[b0045] Harron K., Doidge J.C., Goldstein H. (2020). Assessing data linkage quality in cohort studies. Ann. Hum. Biol..

[b0050] Hendriks N, Koeneman, M.M., van de Sande, A.J.M., Penders, C.G.J., Piek, J.M.J., Kooreman, L.F.S., van Kuijk, S.M.J., Hoosemans, L., Sep, S.J.S., de Vos van Steenwijk, P.J., van Beekhuizen, H.J., Slangen, B.F.M., Nijman, H.W., Kruitwagen, R.F.P.M., Kruse, A. Topical Imiquimod Treatment of High-grade Cervical Intraepithelial Neoplasia (TOPIC-3): A Nonrandomized Multicenter Study. Journal of Immunotherapy. 2022;45(3):180-86. 10.1097/CJI.0000000000000414 PMC8906243 35180719

[b0055] Ivers N., Grimshaw J., Jamtvedt G., Flottorp S., O’Brien M., French S., Young J., Odgaard-Jensen J. (2014). Growing literature, stagnant science? systematic review, meta-regression and cumulative analysis of audit and feedback interventions in health care. J. Gen. Intern. Med..

[b0060] Kyrgiou M., Athanasiou A., Paraskevaidi M., Mitra A., Kalliala I., Martin-Hirsch P., Arbyn M., Bennett P., Paraskevaidis E. (2016). Adverse obstetric outcomes after local treatment for cervical preinvasive and early invasive disease according to cone depth: systematic review and meta-analysis. Br. Med. J..

[b0065] Loopik D.L., Siebers A.G., Melchers W.J.G., Massuger F.A.G., Bekkers R.L.M. (2020). Clinical practice variation and overtreatment risk in women with abnormal cervical cytology in the Netherlands: two-step versus see-and-treat approach. Am. J. Obstetr. Gynaecol..

[b0070] Loopik D.L., Bekkers R.L.M., van Drongelen J., Voorham Q.J.M., Melchers W.J.G., Massuger L.F.A.G., van Kemenade F.J., Siebers A.G. (2021). Cervical intraepithelial neoplasia and the risk of spontaneous preterm birth: A Dutch population-based cohort study with 45,259 pregnancy outcomes. PLos Med..

[b0075] McCredie M.R.E., Sharples K.J., Paul C., Baranyai J., Medley G., Jones R.W., Skegg D.C.G. (2008). Natural history of cervical neoplasia and risk of invasive cancer in women with cervical intraepithelial neoplasia 3: a retrospective cohort study. Lancet Oncol..

[b0080] Mercuri M., Gafni A. (2017). Examining the role of the physician as a source of variation: Are physician-related variations necessarily unwarranted?. J. Eval. Clin. Pract..

[b0085] PALGA, 2021. Stichting PALGA 2021. Available from: https://www.palga.nl/over-ons/stichting-palga/.

[b0090] Pimple S., Mishra G. (2022). Cancer cervix: epidemiology and disease burden. CytoJ..

[b0095] The American College of Obstetricians and Gynecologists. Choosing Wisely 2016. Available from: https://www.acog.org/practice-management/patient-safety-and-quality/partnerships/choosing-wisely.

[b0100] Vektis. Over Vektis 2021. Available from: https://www.vektis.nl/over-vektis.

[b0105] Vogel J., Chawanpaiboon S., Moller A., Watananirun K., Bonet M., Lumbiganon P. (2018). The global epidemiology of preterm birth. Best Pract. Res. Clin. Obstetr. Gynaecol..

[b0110] Walboomers J., Jacobs M.V., Manos M.M., Bosch F.X., Kummer J.A., Shah K.V., Snijders P.J.F., Peto J., Meijer C.J.L.M., Munoz N. (1999). Human papillomavirus is a necessary cause of invasive cervical cancer worldwide. J. Pathol..

[b0115] Watanabe T., Mikami M., Katabuchi H., Kato S., Kaneuchi M., Takahashi M., Nakai H., Nagase S., Niikura H., Mandai M., Hirashima Y., Yanai H., Yamagami W., Kamitani S., Higashi T. (2018). Quality indicators for cervical cancer care in Japan. J. Gynecol. Oncol..

[b0120] Wennberg J.E. (2002). Unwarranted variations in healthcare delivery: implications for academic medical centres. Br. Med. J..

[b0125] Wennberg J.E. (2002). Unwarranted variations in healthcare delivery: implications for academic medical centres. Br. Med. J..

[b0130] Zorg TTP. Zorg TTP 2021. Available from: https://www.zorgttp.nl/.

[b0135] Zorginstituut Nederland. Verbetersignalement Baarmoederhalsafwijking CIN. Zorginstituut Nederland; 2019 24th September 2019.

[b0140] Zorginstituut Nederland. Baarmoederhalsafwijkingen 2021. Available from: https://www.zorginzicht.nl/kwaliteitsinstrumenten/baarmoederhalsafwijkingen.

