# Supplementary material for: Identification of over- and undertreatment in the Dutch national cervical cancer screening program: A data linkage study at the hospital level
Source: Prev Med Rep. 2023 Feb 10;32:102134. doi: 10.1016/j.pmedr.2023.102134 (PMC9958351; doi:10.1016/j.pmedr.2023.102134)
Supplement: Supplementary Appendix A — Data definitions [file mmc1.docx]

**Appendix A: Data definitions**

| n (%) |
| --- |
| Age, y, median (range)^[[1]](#footnote-1)^ |
| Hr-HPV status, n (%)^[[2]](#footnote-2)^ |
| - Negative |
| - Positive |
| - Unknown / invalid |
| Referral cytology, n (%)^[[3]](#footnote-3)^ |
| - Low-grade |
| - High-grade |
| Histologic results from biopsy, n (%) |
| - CIN < 1^[[4]](#footnote-4)^ |
| - CIN 2^[[5]](#footnote-5)^ |
| - CIN 3^[[6]](#footnote-6)^ |

Table 1: Baseline characteristics of study population

| Final histological results from treatment specimen, n (%) |
| --- |
| - CIN < 1^[[7]](#footnote-7)^ |
| - CIN 2^[[8]](#footnote-8)^ |
| - CIN > 3^[[9]](#footnote-9)^ |
| - Other |
| Number of hospitals |
| Patients per hospital |
| Colposcopies^[[10]](#footnote-10)^ per hospital |
| LEEPs^[[11]](#footnote-11)^ per hospital |
| CNKs^[[12]](#footnote-12)^ per hospital |

Table 1: Baseline characteristics of study population

**Category 1:**

Management strategy: whether the clinician decided to perform a treatment, given specific cytological or histological information.

| **Quality indicator** | **Numerator** | **Denominator** |
| --- | --- | --- |
| 1. See-and-treat low grade cytology | LEEP without biopsy^[[13]](#footnote-13)^ | Patients with low grade cytology^[[14]](#footnote-14)^ that underwent a colposcopy^[[15]](#footnote-15)^ |
| 1. See-and-treat high grade cytology | LEEP without biopsy^13^ | High grade cytology^[[16]](#footnote-16)^ that underwent a colposcopy^3^ |
| 1. Treatment CIN 1 | LEEP within 3 months after biopsy CIN 1^[[17]](#footnote-17)^ | Biopsy result < CIN 1 after colposcopy^[[18]](#footnote-18)^ |
| 1. Treatment CIN 2 | LEEP within 3 months after biopsy CIN 2^[[19]](#footnote-19)^ | Biopsy result CIN 2 after colposcopy^[[20]](#footnote-20)^ |
| 1. Treatment CIN 3 | LEEP within 3 months after biopsy CIN 3^[[21]](#footnote-21)^ | Biopsy result CIN 3 after colposcopy^[[22]](#footnote-22)^ |

Table 2: Definition of quality indicators

**Category 2:**

Outcome of treatment: treatment specimen after colposcopy with biopsy or see-and-treat approach.

| **Quality indicator** | **Numerator** | **Denominator** |
| --- | --- | --- |
| 1. Treatment specimen CIN | Treatment specimen  6.1 CIN < 1 after LEEP  6.2 CIN 2 after LEEP  6.3 CIN > 3 after LEEP^[[23]](#footnote-23)^ | Total of LEEPs performed^[[24]](#footnote-24)^ |
| 1. Normalization rate CIN 2 | No. of patients with < Pap 2 cytology at follow-up after LEEP^[[25]](#footnote-25)^ | No. of patients with treatment specimen CIN 2 after LEEP^[[26]](#footnote-26)^ |
| 1. Normalization rate CIN 3 | No. of patients with < Pap 2 cytology at follow-up after LEEP^25^ | No. of patients with treatment specimen CIN 3 after LEEP^[[27]](#footnote-27)^ |

Table 2: Definition of quality indicators

**Category 3:**

Follow-up: adherence to the Dutch guideline on follow-up after colposcopy with biopsy or treatment.

| **Quality indicator** | **Numerator** | **Denominator** |
| --- | --- | --- |
| 1. Timeframe follow-up cytology after wait-and-see colposcopy with biopsy CIN 1 or low-grade referral cytology without biopsy at colposcopy    1. Between 10-14 months    2. < 10 months    3. > 14 months | No. of patients who had follow-up cytology performed (with or without HPV)^[[28]](#footnote-28)^ | No. of patients with colposcopy (without treatment) and biopsy CIN 1 or low-grade referral cytology without biopsy at colposcopy^[[29]](#footnote-29)^ |
| 1. Timeframe follow-up cytology after LEEP with treatment specimen CIN 2    1. Between 4-8 months    2. < 4 months    3. > 8 months | No. of patients who had follow-up cytology performed (with or without HPV)^28^ | No. of patients who were treated with LEEP^[[30]](#footnote-30)^ and treatment specimen showed CIN 2^[[31]](#footnote-31)^ |
| 1. Timeframe follow-up cytology after wait-and-see colposcopy with biopsy CIN 2    1. Between 10-14 months    2. < 10 months    3. > 14 months | No. of patients who had follow-up cytology performed (with or without HPV)^28^ | No. of patients who underwent colposcopy (without treatment) and biopsy CIN 2^[[32]](#footnote-32)^ |
| 1. Timeframe follow-up cytology after LEEP with treatment specimen CIN 3    1. Between 4-8 months    2. < 4 months    3. > 8 months | No. of patients who had follow-up cytology performed (with or without HPV)^28^ | No. of patients who were treated with LEEP^33^ and treatment specimen showed CIN 3^[[33]](#footnote-33)^ |

Table 2: Definition of quality indicators

1. Age based on PALGA dataset at date of first observation. [↑](#footnote-ref-1)
2. If one observation of a patient shows a HPV positive result, the patients is scored as HPV positive. [↑](#footnote-ref-2)
3. If cervix cytology is followed by a colposcopy (claim code 039170) or LEEP (code 037200) or conization (code 037210) within 2 months after date of receival of cervix cytology, it is considered as referral cytology. [↑](#footnote-ref-3)
4. PALGA diagnosis code diagklasse = N, O, P, V and W were used to identify CIN < 1 and PALGA technique code B was used to identify a biopsy. When within a timeframe of 7 days before the date of registration of the biopsy in the PALGA dataset a LEEP or conization was registered in the Vektis dataset (claim code claim code 037200 or 037210), the observation was excluded as it was unsure whether a biopsy or LEEP / conization was performed. [↑](#footnote-ref-4)
5. PALGA diagnosis code diagklasse = M was used to identify CIN 2 and PALGA technique code B was used to identify a biopsy. When within a timeframe of 7 days before the date of registration of the biopsy in the PALGA dataset a LEEP or conization was registered in the Vektis dataset (claim code claim code 037200 or 037210), the observation) was excluded, as it was unsure whether a biopsy or LEEP / conization was performed. [↑](#footnote-ref-5)
6. PALGA diagnosis code diagklasse = I, J, K and L were used to identify CIN 3 and PALGA technique code B was used to identify the biopsy. When within a timeframe of 7 days before the date of registration of the biopsy in the PALGA dataset a LEEP or conization was registered in the Vektis dataset (claim code claim code 037200 or 037210), the observation was excluded as it was unsure whether a biopsy or LEEP / conization was performed. CIN 3 includes adenocarcinoma in situ (AIS). [↑](#footnote-ref-6)
7. PALGA diagnosis code diagklasse = N, O, P, V and W were used to identify CIN < 1. The observation should be defined as a LEEP or conization in both the datasets, Vektis (claim code 037200 or 037210) and PALGA (technique code A). If the observation was only defined as LEEP or conization in one of the two datasets, the observation was removed. When the LEEP or conization observation was registered in the PALGA dataset within 7 days after it was registered in the Vektis dataset, it was considered as the same observation. [↑](#footnote-ref-7)
8. PALGA diagnosis code diagklasse = M was used to identify CIN 2. The observation should be defined as a LEEP or conization in both the datasets, Vektis (claim code 037200 or 037210) and PALGA (technique code A). If the observation was only defined as LEEP or conization in one of the two datasets, the observation was removed. When the LEEP or conization observation was registered in the PALGA dataset within 7 days after it was registered in the Vektis dataset, it was considered as the same observation. [↑](#footnote-ref-8)
9. PALGA diagnosis code diagklasse = ABCDEGIJKLT1 were used to identify CIN 3. The observation should be defined as a LEEP or conization in both the datasets, Vektis (claim code 037200 or 037210) and PALGA (technique code A). If the observation was only defined as LEEP or conization in one of the two datasets, the observation was removed. When the LEEP or conization observation was registered in the PALGA dataset within 7 days after it was registered in the Vektis dataset, it was considered as the same observation. CIN 3 includes adenocarcinoma in situ (AIS). [↑](#footnote-ref-9)
10. Based on Vektis claim code 039170. When within a timeframe of 7 days after the date of registration of the colposcopy a LEEP was registered in the PALGA dataset (technique code A), the observation was excluded, as it was unsure whether a colposcopy or see-and-treat procedure was performed. [↑](#footnote-ref-10)
11. The observation should be defined as a LEEP in both the datasets, Vektis (claim code 037200) and PALGA (technique code A). If the observation was only defined as LEEP or conization in one of the two datasets, the observation was removed. When the LEEP or conization observation was registered in the PALGA dataset within 7 days after it was registered in the Vektis dataset, it was considered as the same observation. [↑](#footnote-ref-11)
12. The observation should be defined as a conization in both the datasets, Vektis (claim code 037210) and PALGA (technique code A). If the observation was only defined as conization in one of the two datasets, the observation was removed. When the conization observation was registered in the PALGA dataset within 7 days after it was registered in the Vektis dataset, it was considered as the same observation. [↑](#footnote-ref-12)
13. The first observation in the PALGA dataset after cervix cytology should be a LEEP or conization. The observation should be defined as a LEEP or conization in both the datasets, Vektis (claim code 037200 or 037210) and PALGA (technique code A). If the observation was only defined as LEEP or conization in one of the two datasets, the observation was removed. When the LEEP or conization observation was registered in the PALGA dataset within 7 days after it was registered in the Vektis dataset, it was considered as the same observation. [↑](#footnote-ref-13)
14. Atypical squamous cells of undetermined significance [ASC-US] or low-grade squamous intraepithelial lesion [LSIL]), identified by PALGA diagnosis code diagkl = b and advieskl = a. [↑](#footnote-ref-14)
15. The colposcopy should be claimed and therefore registered in the Vektis dataset (claim code 039170). When only a LEEP or conization (code 037200 or 037210), without colposcopy was claimed, it was assumed that also a colposcopy was performed. If no interval existed between (referral) cytology and colposcopy or LEEP/conization, patients were excluded. [↑](#footnote-ref-15)
16. High-grade squamous intraepithelial lesion [HSIL] or more, identified by PALGA diagnosis codes diagkl = a and advieskl = a [↑](#footnote-ref-16)
17. The observation should be defined as a LEEP or conization in both the datasets, Vektis (claim code 037200 or 037210) and PALGA (technique code A). If the observation was only defined as LEEP or conization in one of the two datasets, the observation was removed. PALGA diagnosis code diagklasse = N, O, P and technique code B were used to identify biopsy < CIN 1. [↑](#footnote-ref-17)
18. Patients for whom in PALGA a biopsy of the cervix was registered (technique code “B”) with diagnosis code diagklasse = N, O, P, for < CIN 1. When within a timeframe of 7 days before the date of registration of the biopsy in the PALGA dataset a LEEP or conization was registered in the Vektis dataset (claim code claim code 037200 or 037210), the observation was excluded as then it was unknown whether see-and-treat approach was performed instead of a two-step approach. Patients with a hysterectomy after biopsy were removed. [↑](#footnote-ref-18)
19. The observation should be defined as a LEEP or conization in both the datasets, Vektis (claim code 037200 or 037210) and PALGA (technique code A). If the observation was only defined as LEEP or conization in one of the two datasets, the observation was removed. PALGA diagnosis codes diagklasse = M and technique code B were used to identify biopsy CIN 2. [↑](#footnote-ref-19)
20. Patients for whom in PALGA a biopsy of the cervix was registered (technique code “B”) with diagnosis code diagklasse = M for CIN 2. When within a timeframe of 7 days before the date of registration of the biopsy in the PALGA dataset a LEEP or conization was registered in the Vektis dataset (claim code claim code 037200 or 037210), the observation was excluded as then it was unknown whether see-and-treat approach was performed instead of a two-step approach. Patients with a hysterectomy after biopsy were removed. [↑](#footnote-ref-20)
21. The observation should be defined as a LEEP or conization in both the datasets, Vektis (claim code 037200 or 037210) and PALGA (technique code A). If the observation was only defined as LEEP or conization in one of the two datasets, the observation was removed. PALGA diagnosis code diagklasse = I, J, K and L for CIN 3 and technique code B were used to identify biopsy CIN 3. CIN 3 includes adenocarcinoma in situ (AIS). [↑](#footnote-ref-21)
22. Patients for whom in PALGA a biopsy of the cervix was registered (technique code “B”) with diagnosis code I, J, K, L for CIN 3. When within a timeframe of 7 days before the date of registration of the biopsy in the PALGA dataset a LEEP or conization was registered in the Vektis dataset (claim code claim code 037200 or 037210), the observation (patient) was excluded as then it was unknown whether see-and-treat approach was performed instead of a two-step approach. Patients with a hysterectomy after biopsy were removed. CIN 3 includes adenocarcinoma in situ (AIS). [↑](#footnote-ref-22)
23. The observation should be defined as a LEEP or conization in both the datasets, Vektis (claim code 037200 or 037210) and PALGA (technique code A). If the observation was only defined as LEEP or conization in one of the two datasets, the observation was removed. PALGA diagnosis code diagklasse = N, O, P, V and W were used to identify < CIN 1, code diagklasse = M for CIN 2 and code diagklasse = ABCDEGIJKLT1 for CIN 3. CIN 3 includes adenocarcinoma in situ (AIS). [↑](#footnote-ref-23)
24. The observation should be defined as a LEEP or conization in both the datasets, Vektis (claim code 037200 or 037210) and PALGA (technique code A). If the observation was only defined as LEEP or conization in one of the two datasets, the observation was removed. [↑](#footnote-ref-24)
25. PALGA diagnosis codes c, e, g and ( b with diagnosesubcl in (-005, -007, -405,-407, 0116, 0117, 0118, 0119, 0120, 0125, 0126, 0127, 0129, 0131, 0132, 0133, 0134)) were used to identify patients with Pap < 2. [↑](#footnote-ref-25)
26. The observation should be defined as a LEEP or conization in both the datasets, Vektis (claim code 037200 or 037210) and PALGA (technique code A). If the observation was only defined as LEEP or conization in one of the two datasets, the observation was removed. Patients for whom in PALGA a LEEP or CNK of the cervix was registered (technique code “A”) with diagnosis code diagklasse = M for CIN 2. [↑](#footnote-ref-26)
27. The observation should be defined as a LEEP or conization in both the datasets, Vektis (claim code 037200 or 037210) and PALGA (technique code A). If the observation was only defined as LEEP or conization in one of the two datasets, the observation was removed. Patients for whom in PALGA a LEEP or CNK of the cervix was registered (technique code “A”) with diagnosis code diagklasse = I, J, K and L for CIN 3. CIN 3 includes adenocarcinoma in situ (AIS). [↑](#footnote-ref-27)
28. PALGA diagnosis codes a, b, c, d, e were used to identify patients for whom cervix-cytology was performed. [↑](#footnote-ref-28)
29. Patients were included for whom a colposcopy was registered in the Vektis database (code 039170) without a registration of a LEEP or conization on the same day (claim code 037200 or 037210). If in the PALGA dataset a LEEP or conization was registered within 7 days after the colposcopy (PALGA technique code “A”), the patient was excluded. In PALGA diagnosis code = N, O, P, V and W and technieksk = B was used to identify patients with biopsy < CIN 1. Patients without biopsy were included when after 90 days of registration date of the colposcopy no cytological or histological result was registered in the PALGA dataset and their referral cytology was < Pap 2/3a1 (PALGA diagnosis code “a”). When a patient underwent more than one colposcopy, only the first colposcopy was included. [↑](#footnote-ref-29)
30. The observation should be defined as a LEEP or conization in both the datasets, Vektis (claim code 037200 or 037210) and PALGA (technique code M). If the observation was only defined as LEEP or conization in one of the two datasets, the observation was removed. [↑](#footnote-ref-30)
31. PALGA diagnosis code diagklasse = M. [↑](#footnote-ref-31)
32. Patients were included for whom a colposcopy was registered in the Vektis database (code 039170) without a registration of a LEEP or conization (claim code 037200 or 037210) on the same day. If in the PALGA dataset a LEEP or conization was registered within 7 days after the colposcopy (PALGA technique code “B”), the patient was excluded. When a patient underwent more than one colposcopy, only the first colposcopy was included. [↑](#footnote-ref-32)
33. The observation should be defined as a LEEP in both the datasets, Vektis (claim code 037200 or 037210) and PALGA (technique code “A”). If the observation was only defined as LEEP in one of the two datasets, the observation was removed. PALGA diagnosis code diagklasse = I, J, K and L was used to identified treatment specimen CIN 3. CIN 3 includes adenocarcinoma in situ (AIS). [↑](#footnote-ref-33)
