# Supplementary material for: Identification of over- and undertreatment in the Dutch national cervical cancer screening program: A data linkage study at the hospital level
Source: Prev Med Rep. 2023 Feb 10;32:102134. doi: 10.1016/j.pmedr.2023.102134 (PMC9958351; doi:10.1016/j.pmedr.2023.102134)
Supplement: Supplementary Appendix B — Data linkage procedure [file mmc2.docx]

**Appendix B: Data linkage procedure**

|  | Vektis (number of patients) | PALGA (number of patients) |
| --- | --- | --- |
| Raw dataset | 125,751 | 1,699,878 |
| No. of exclusions per step: |  |  |
| 1) Vektis: Patients with similar or absent postal code and/or date of birth | 1,229 |  |
| 2) Zorg TTP: Pre-match procedure | 7,831 | 1,572,445 |
| 3) Patients with one pseudonym in Vektis dataset, corresponding to multiple PALGA patient ID’s | 203 | 101 |
| 4) Pseudonyms with normal cytology, if two similar pseudonyms exist with one normal and one abnormal cytology | - | 10,544 |
| 5) Patients with similar pseudonym and both normal or both abnormal cytology | 478 | 778 |
| 6) Pseudonyms that could not be linked to a hospital | 111 | 111 |
|  |  |  |
| Number of patients that could be linked | 115,899 | 115,899 |
|  |  |  |
| 7) Exclusion of patients treated in hospitals < 500 patients per year | 3,019 | 3,019 |
|  |  |  |
| Final dataset | 112,880 | 112,880 |

Additional information per step of the data-linkage procedure:

Step 1: The postal code in the Vektis dataset was noted at the date of opening DTC G 19.

Step 2: Patients were removed from the datasets if no similar pseudonym was present in both datasets.

Step 3: The patients’ postal code might not correspond between both datasets. To minimize the risk of an incorrect match of data, pseudonyms that matched multiple PALGA patient IDs were removed from both datasets.

Step 4: One pseudonym in the Vektis dataset could correspond with multiple patient IDs in the PALGA dataset. This occurs because the PALGA dataset is significantly larger, involving both patients who were and were not referred to the gynaecologist, and therefore contains more patients with similar date of birth and postal code. For two different patients (identified using the unique PALGA patient ID) with a similar pseudonym (birth and postal code), the patient with normal cervix cytology was excluded from the PALGA dataset, assuming that this patient was not referred to the gynaecologist.

Step 5: If one Vektis pseudonym corresponded to two PALGA patient ID’s that had either both normal cervix cytology or both abnormal cervix cytology, no assumption could be made which pseudonym to include. Consequently, we excluded both pseudonyms.
