# Supplementary material for: Identification of over- and undertreatment in the Dutch national cervical cancer screening program: A data linkage study at the hospital level
Source: Prev Med Rep. 2023 Feb 10;32:102134. doi: 10.1016/j.pmedr.2023.102134 (PMC9958351; doi:10.1016/j.pmedr.2023.102134)

**Appendix C: Funnel plots**

Indicator 1 (see-and-treat low grade referral cytology)

Age < 40 years:


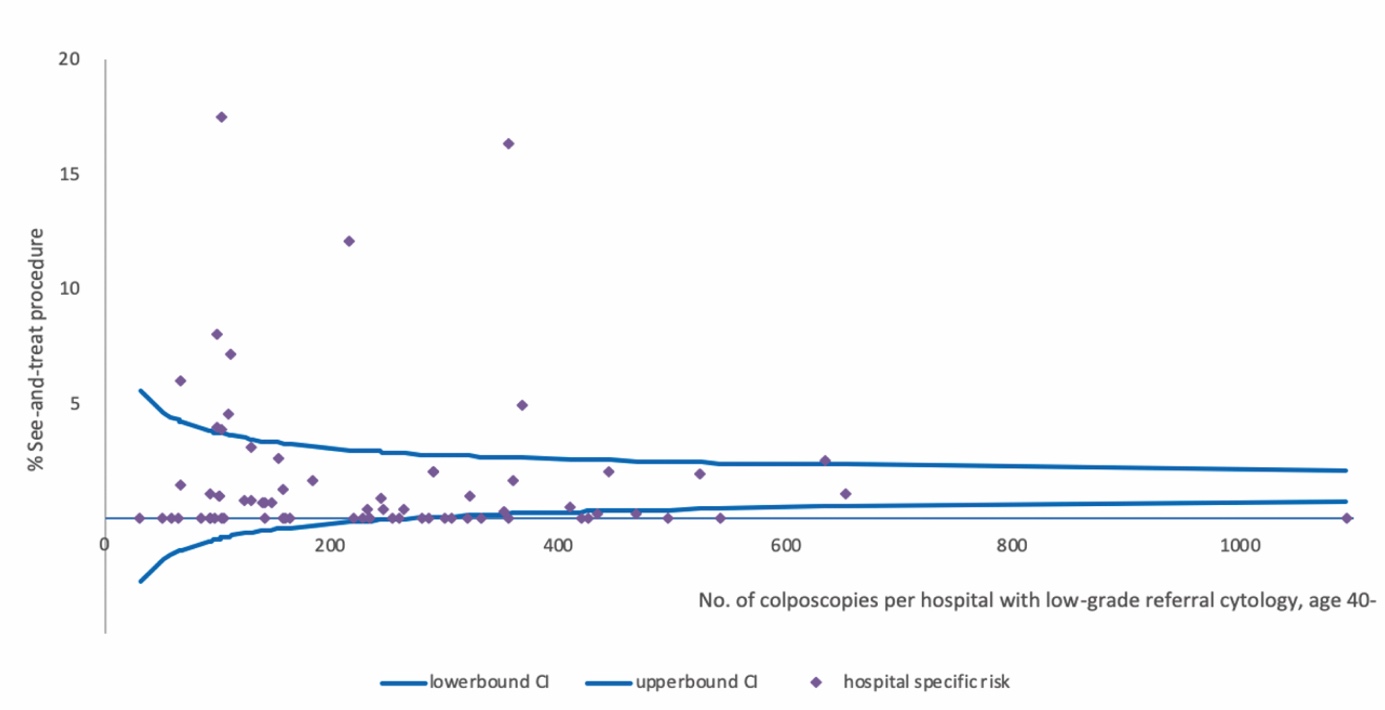


Age > 40 years:


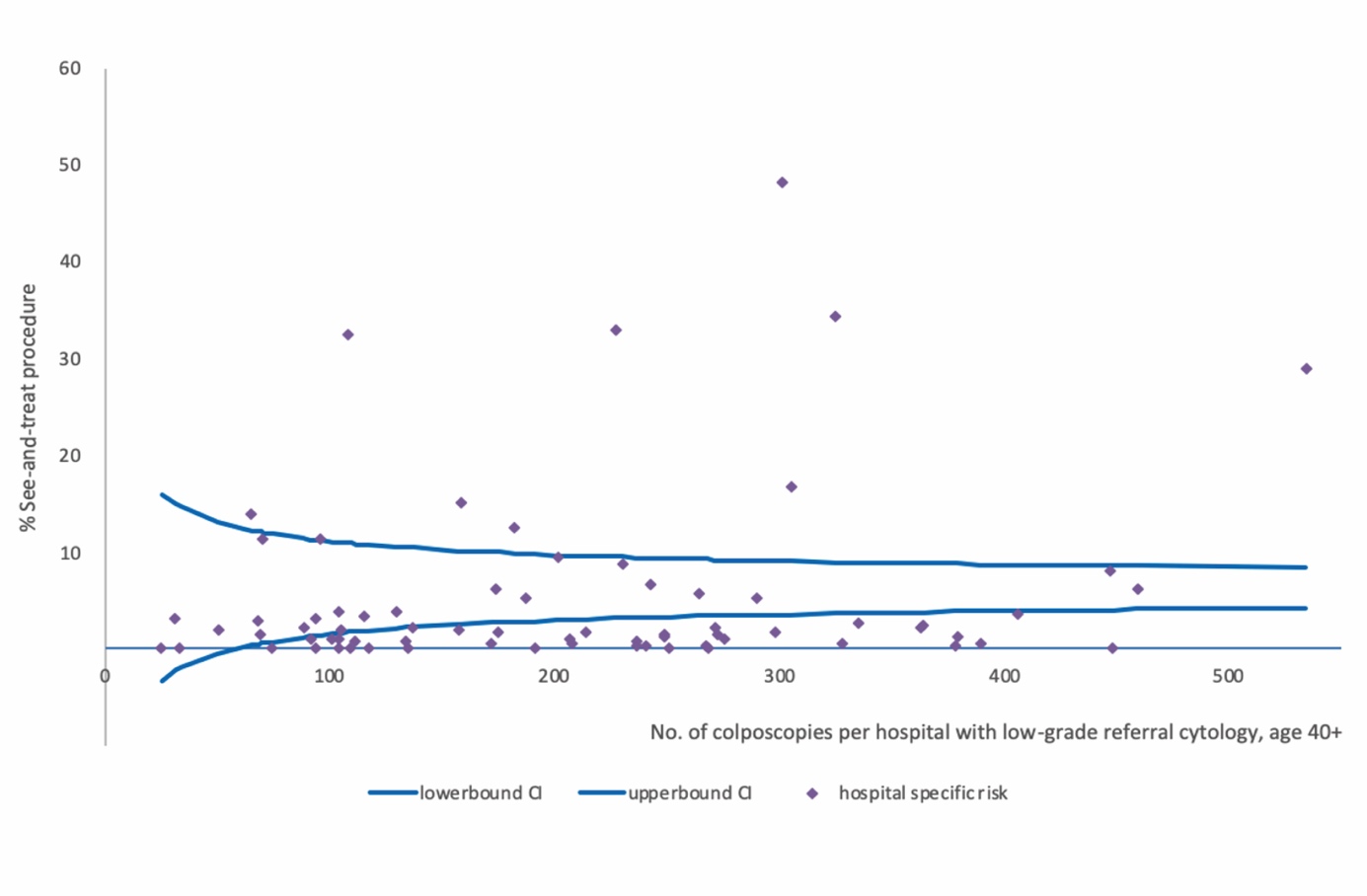


Indicator 2 (see-and-treat high grade referral cytology)

Age < 40 years:


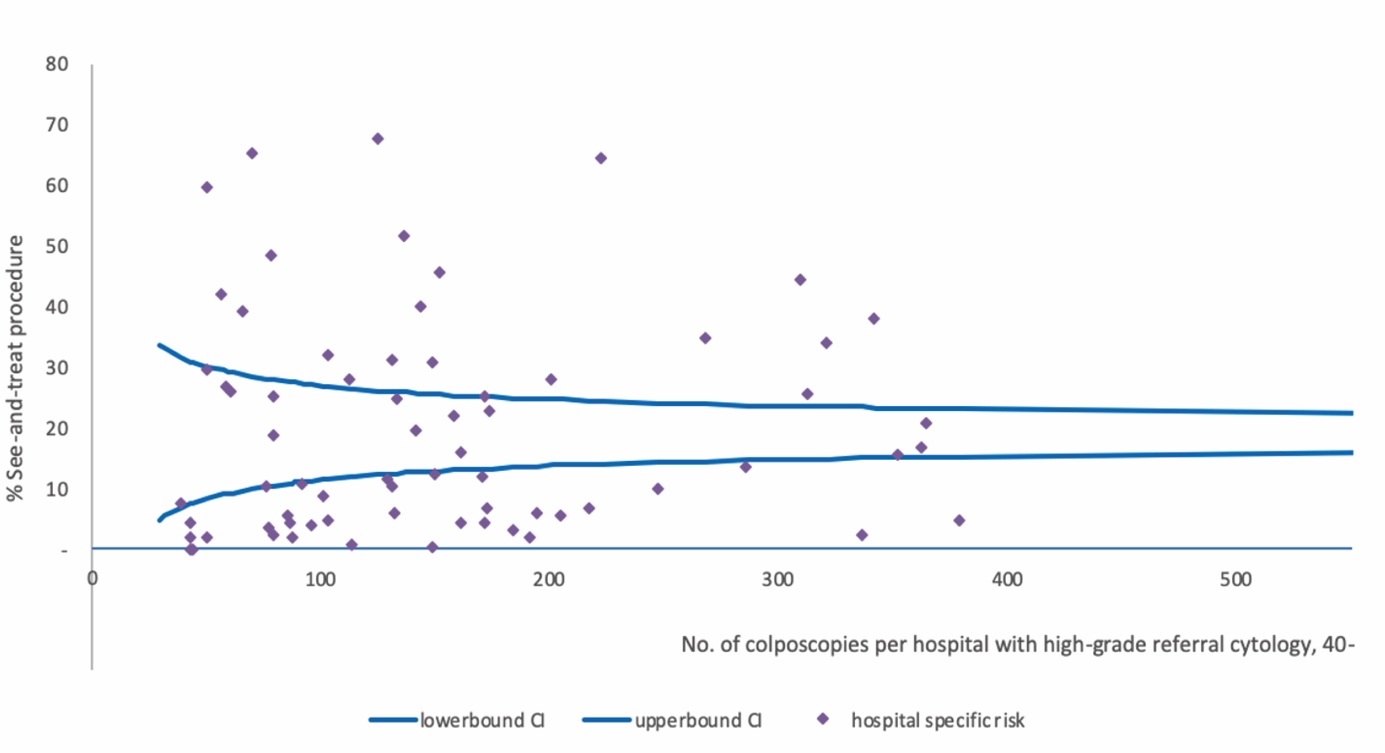


Age > 40 years:


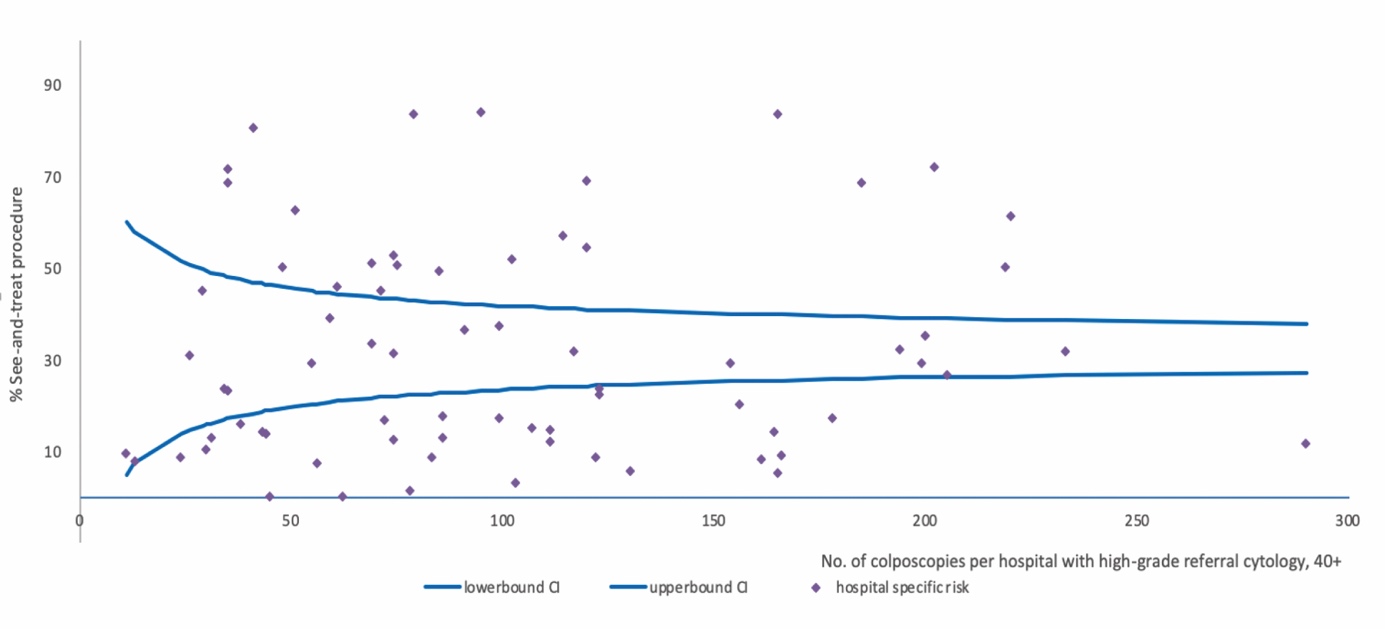


Indicator 3 (Treatment after biopsy CIN 1)

Age < 40 years:


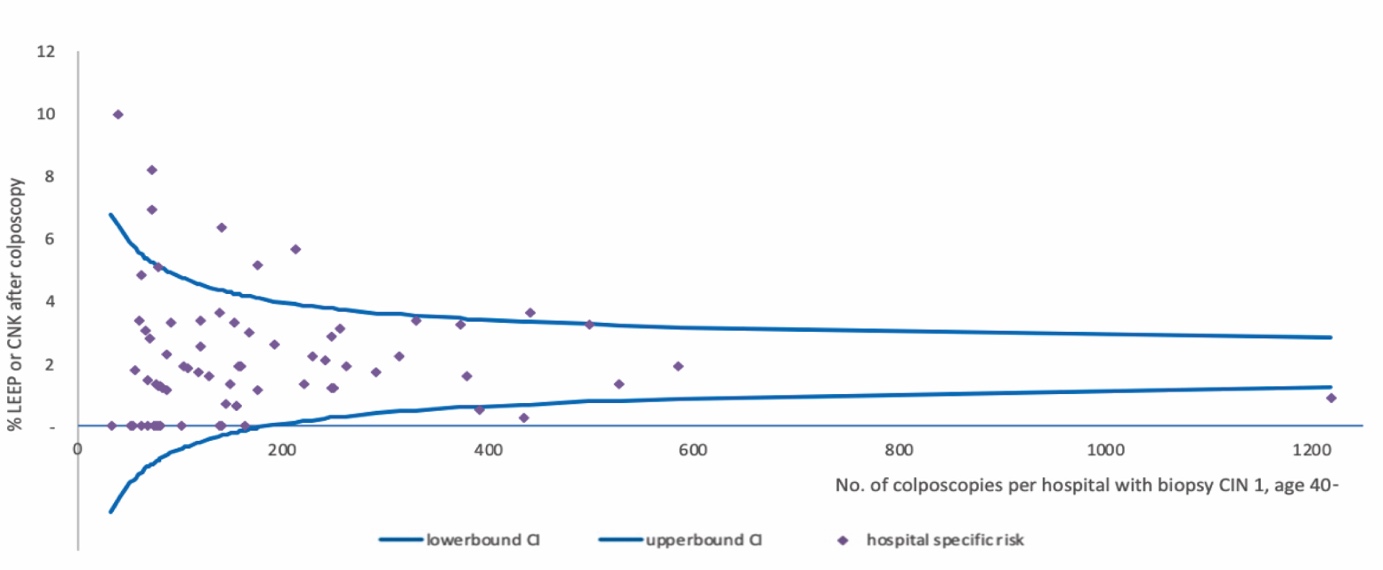


Age > 40 years:


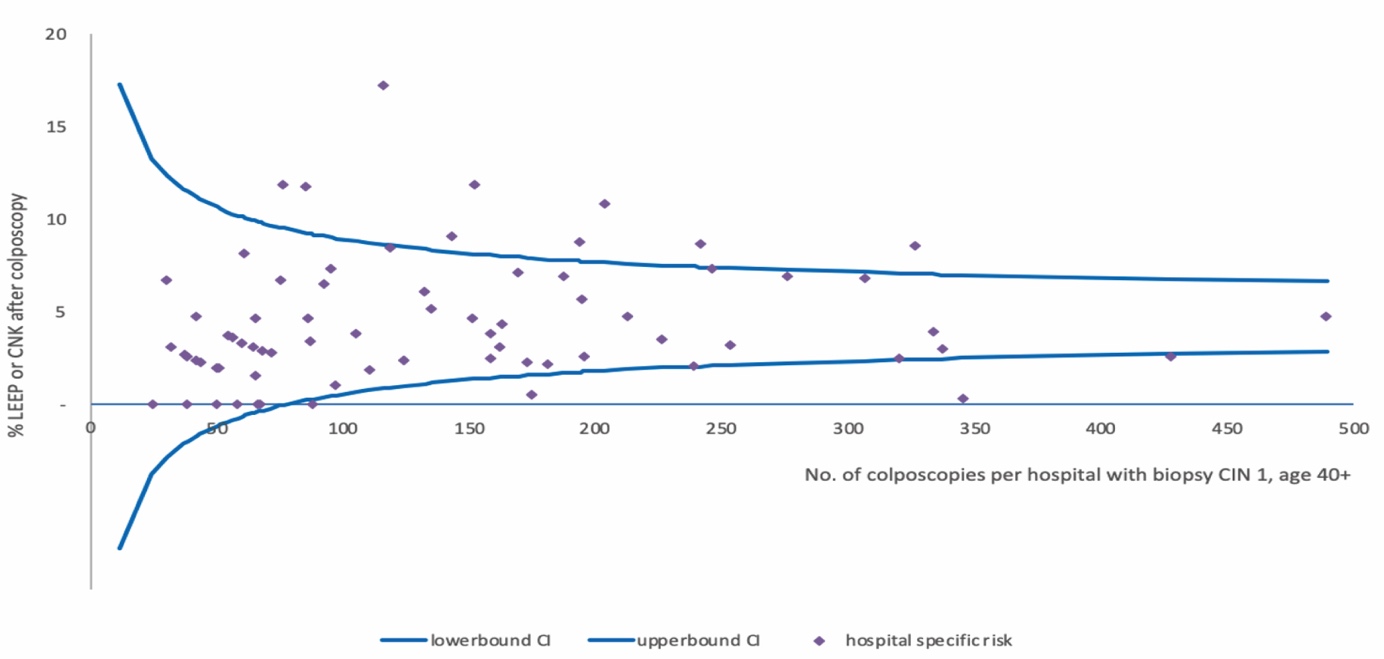


Indicator 4 (Treatment after biopsy CIN 2):

Age < 40 years:


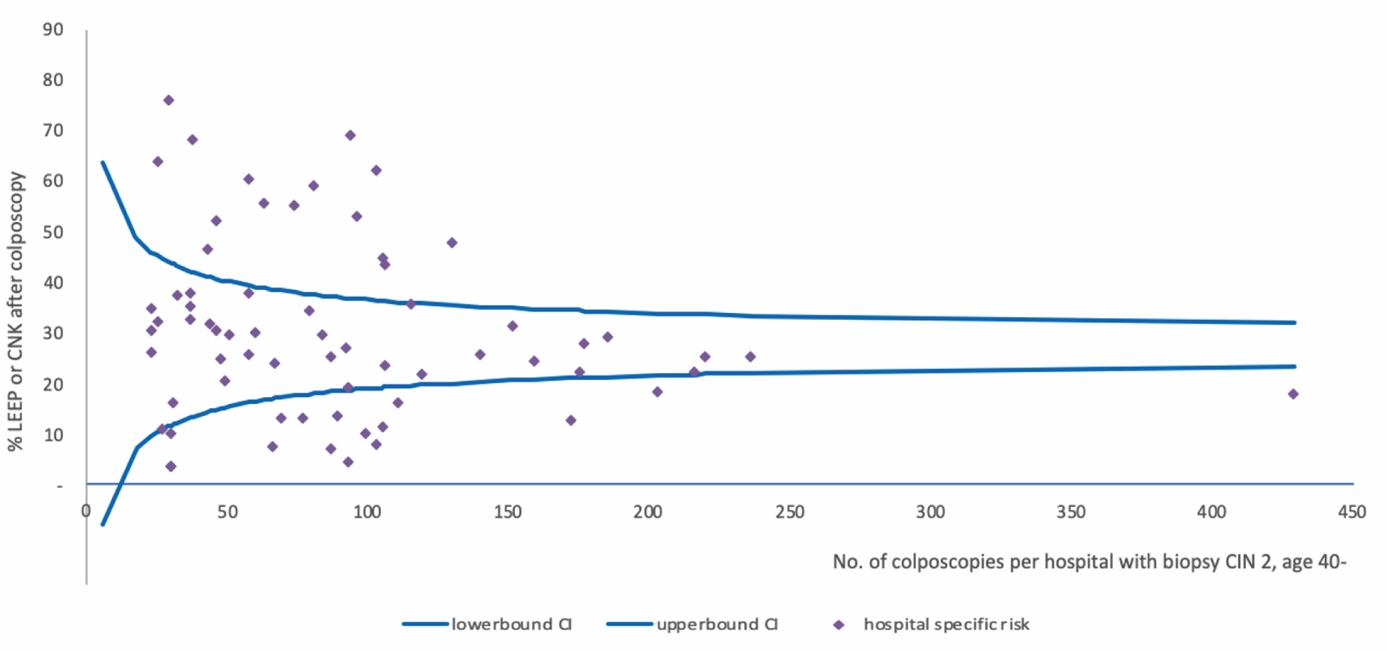


Age > 40 years:


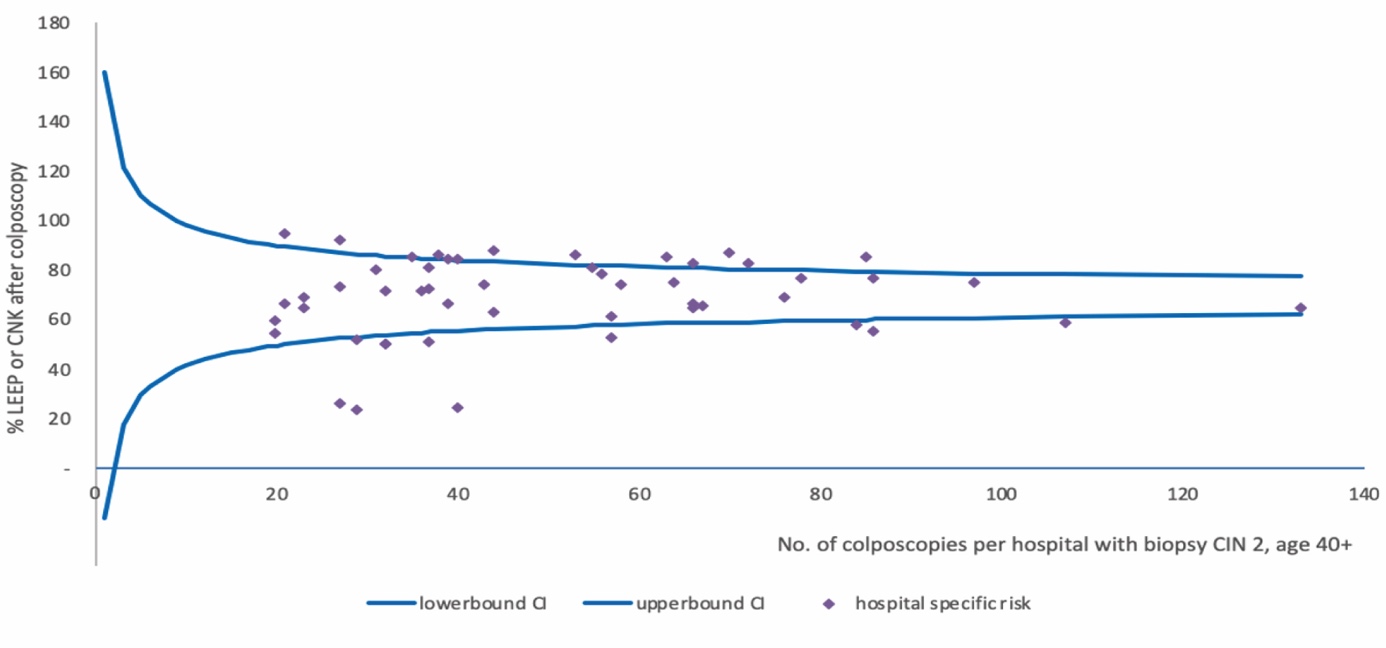


Indicator 5 (Treatment after biopsy CIN 3)


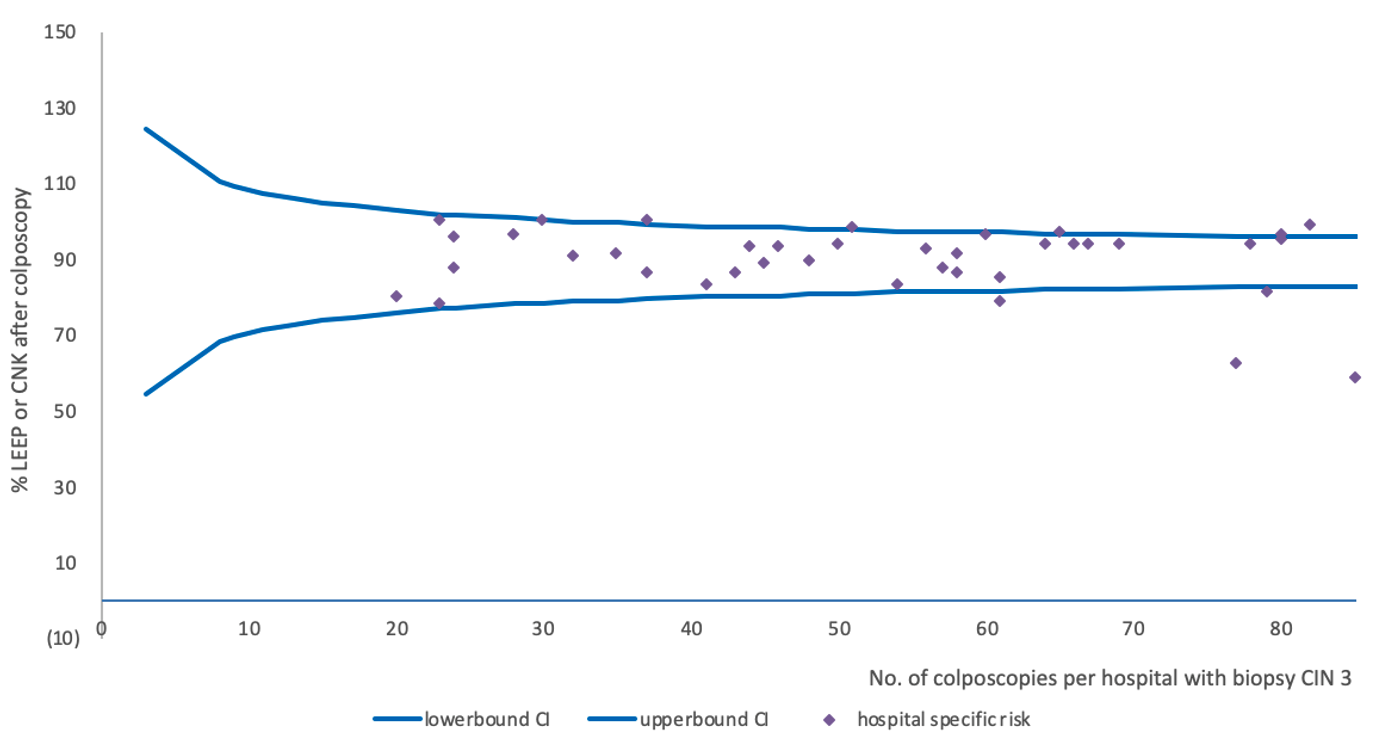


Indicator 6 (Treatment specimen)


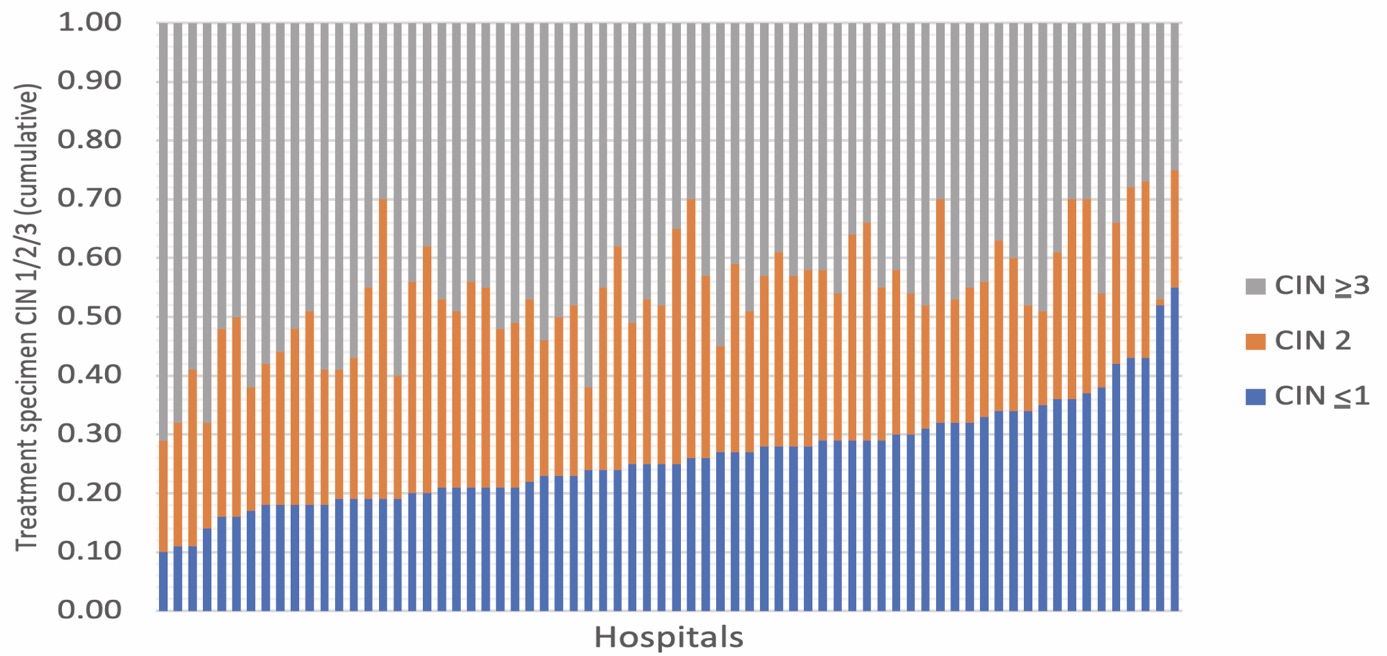


Indicator 7 (Normalization rate CIN 2)


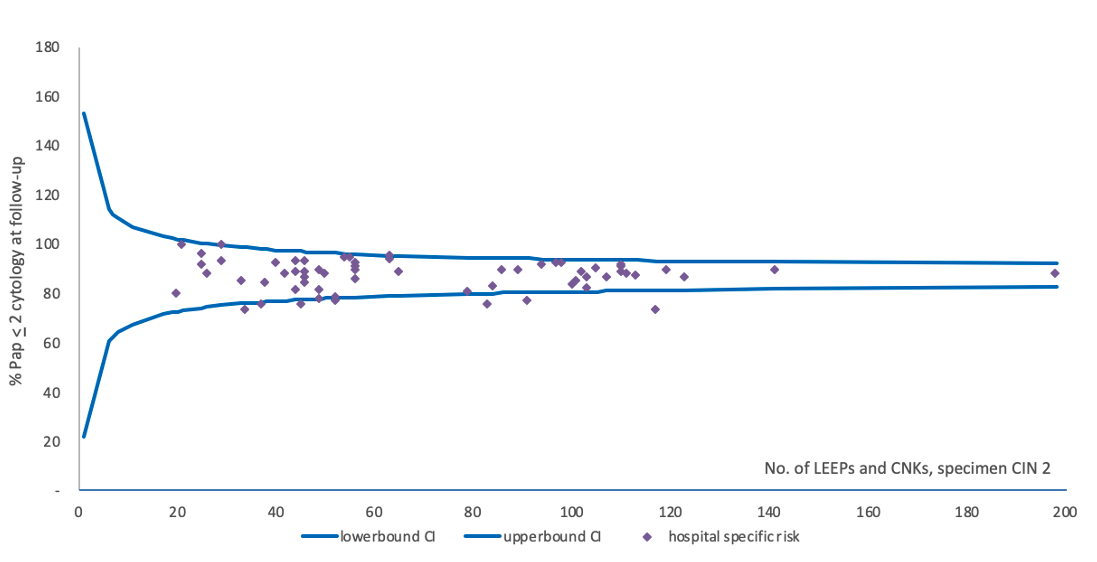


Indicator 8 (Normalization rate CIN 3)


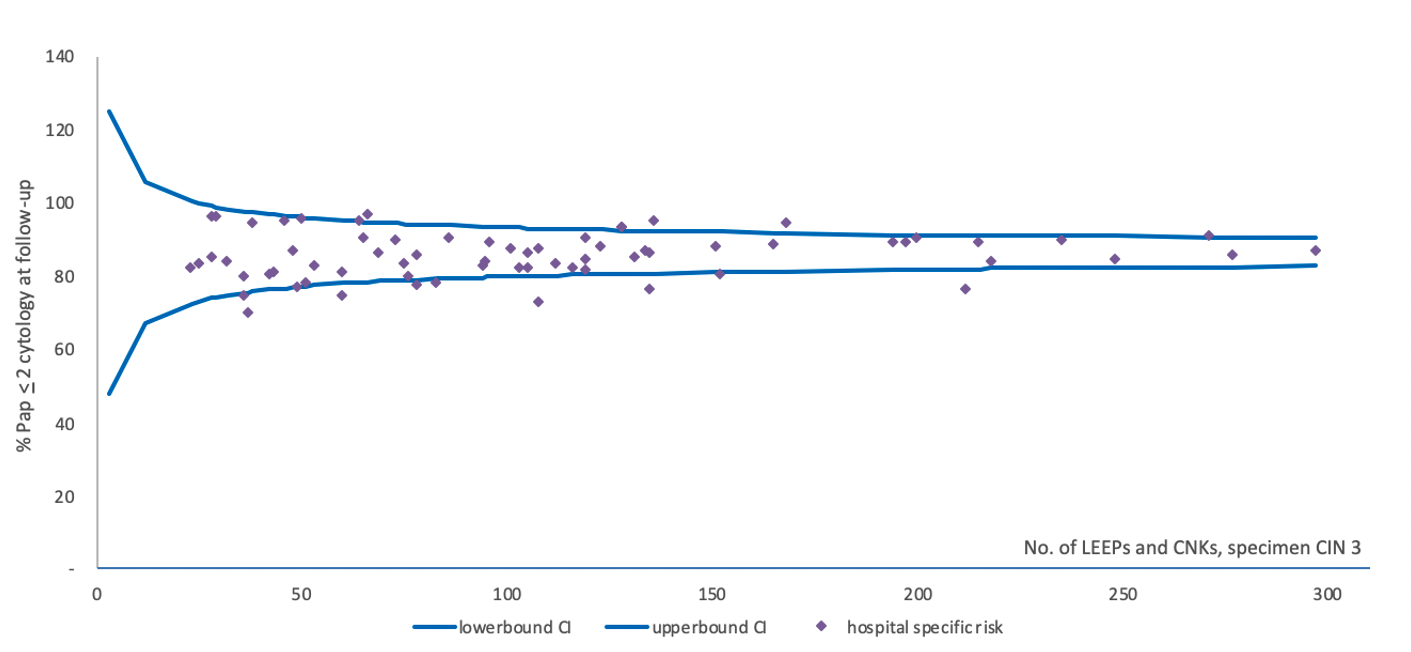


Indicator 9 (Follow-up wait-and-see colposcopy and/or biopsy CIN 1):

Funnel plot shows results for women that had their follow-up received within the correct timeframe (indicator 9.1; between 10-14 months).


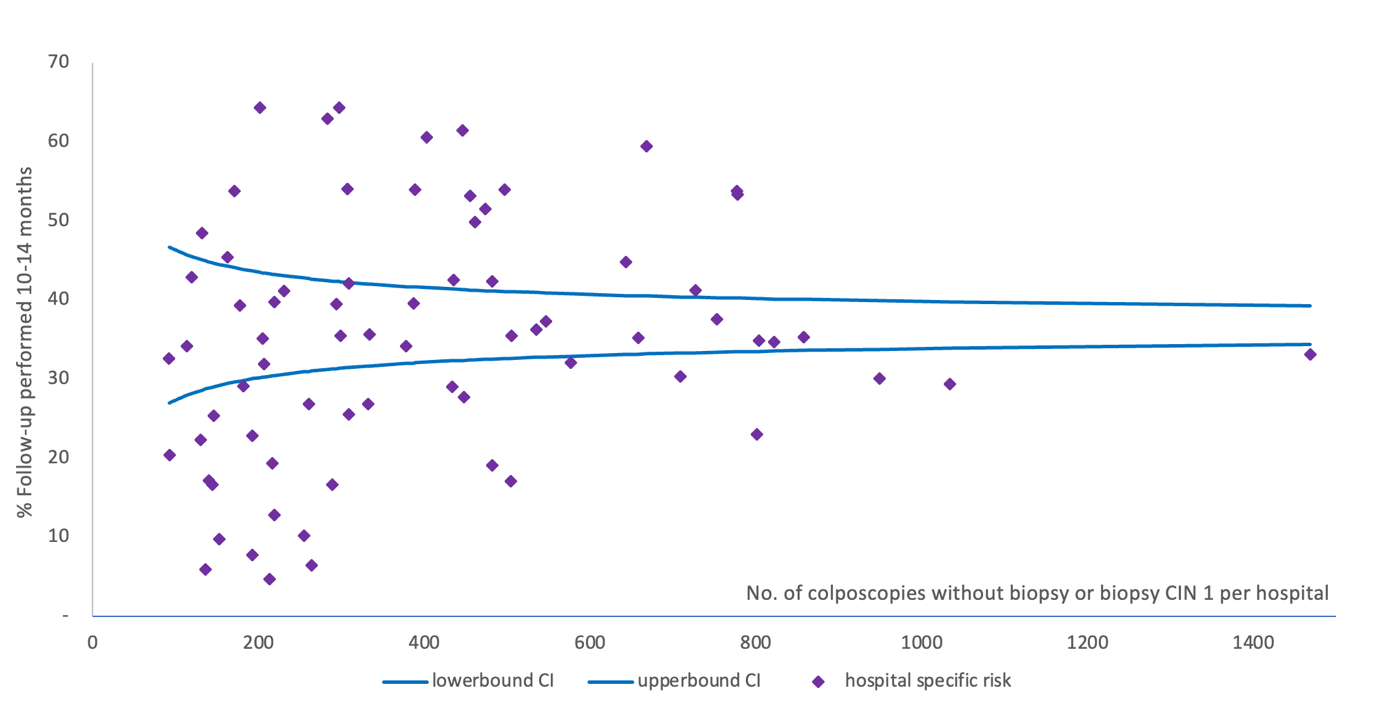


Indicator 10 (Follow-up treatment CIN 2):

Funnel plot shows results for women that had their follow-up received within the correct timeframe (indicator 10.1; between 4-8 months).


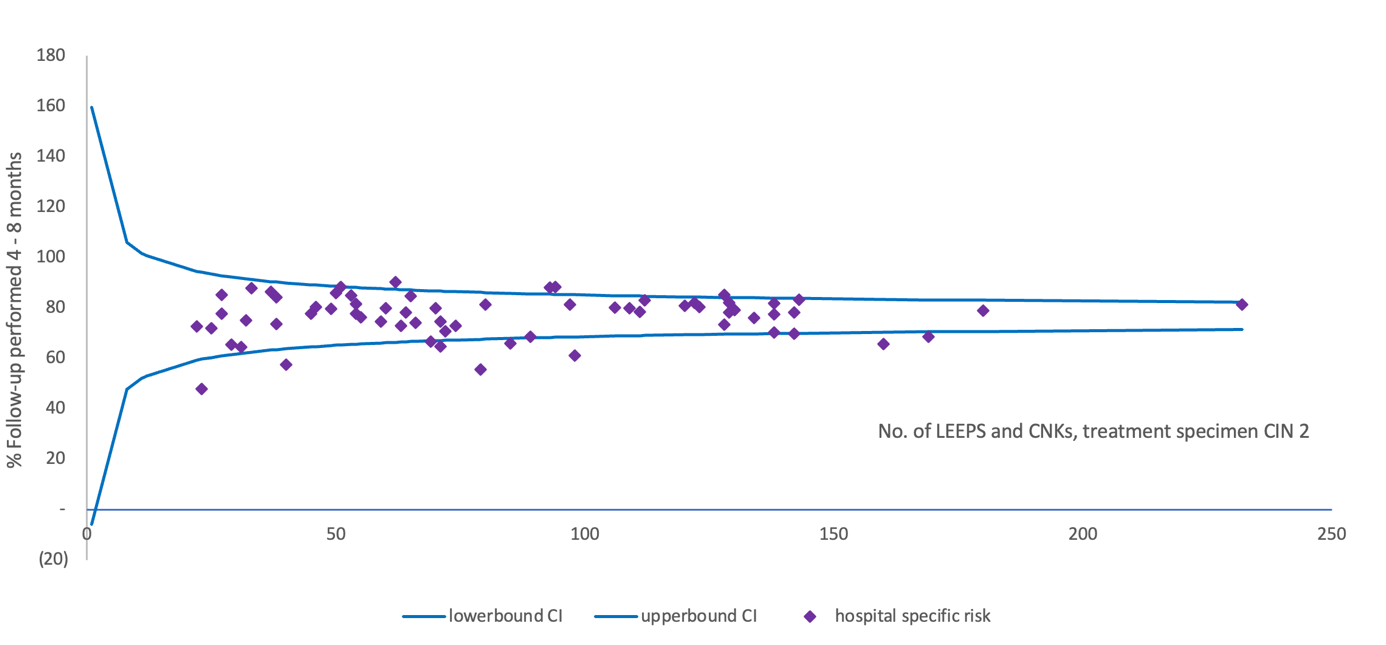


Indicator 11 (Follow-up biopsy CIN 2 without treatment):

Funnel plot shows results for women that had their follow-up received within the correct timeframe (indicator 11.1; between 10-14 months).


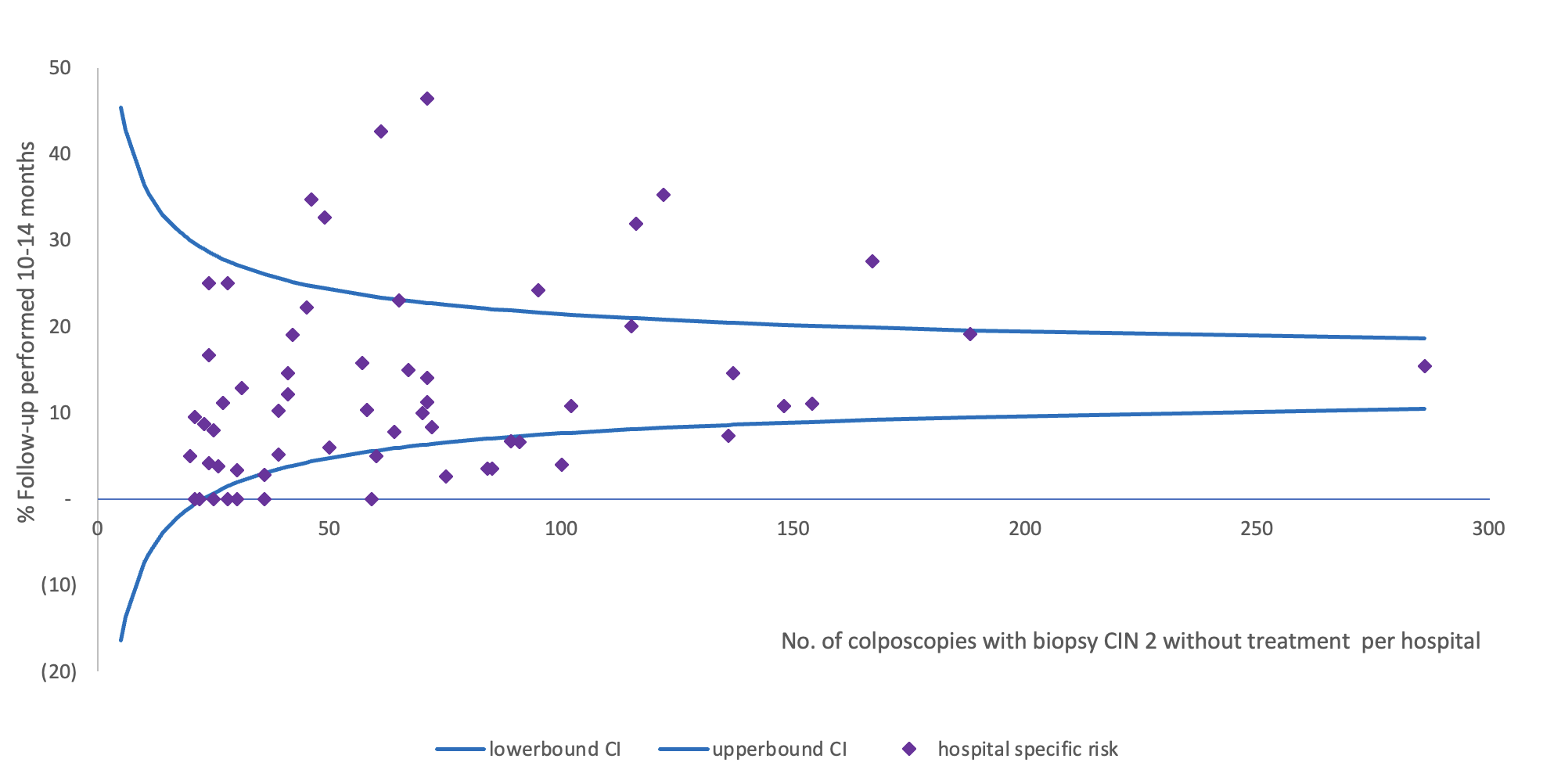


Indicator 12 (Follow-up treatment CIN 3):

Funnel plot shows results for women that had their follow-up received within the correct timeframe (indicator 12.1; between 4-8 months).


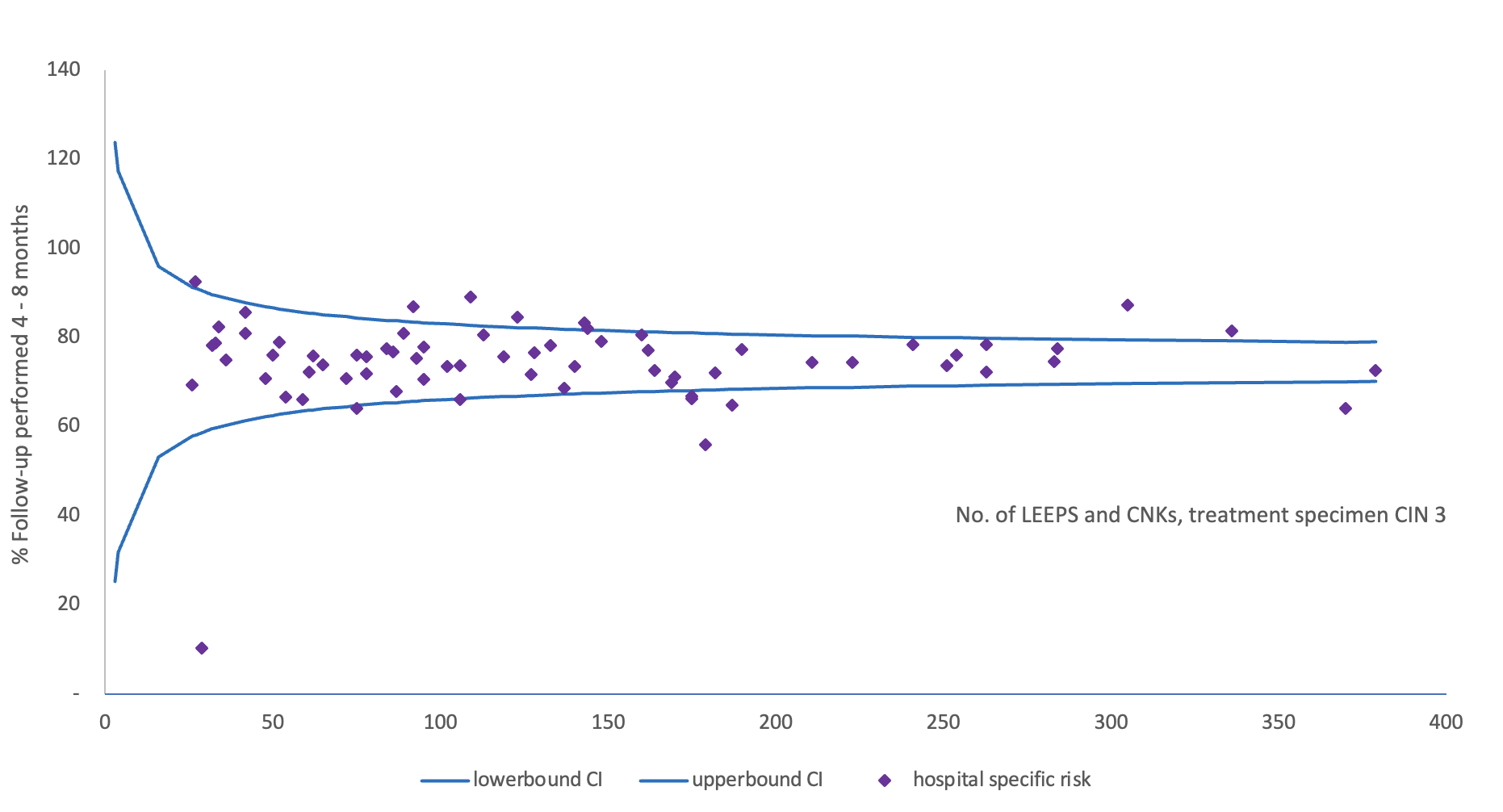

Supplement: Supplementary Appendix C — Funnel plots [file mmc3.docx]
