# Supplementary material for: Identification of over- and undertreatment in the Dutch national cervical cancer screening program: A data linkage study at the hospital level
Source: Prev Med Rep. 2023 Feb 10;32:102134. doi: 10.1016/j.pmedr.2023.102134 (PMC9958351; doi:10.1016/j.pmedr.2023.102134)
Supplement: Supplementary Table A.1 [file mmc5.docx]

**Table A.1: Definition of all indicators**

| **Category** | **Quality indicator** | **Numerator** | **Denominator** | **Source** | **Dutch guideline** | **Purpose indicator** |
| --- | --- | --- | --- | --- | --- | --- |
| Management strategy | 1. See-and-treat low grade cytology | LEEP without biopsy | Patients with low-grade referral cytology that underwent a colposcopy | Dutch guideline^4^ | Do not perform see-and-treat for low grade cytology^[[1]](#footnote-1)^ | Overtreatment |
| Management strategy | 1. See-and-treat high grade cytology^[[2]](#footnote-2)^ | LEEP without biopsy | Patients with high-grade referral cytology that underwent a colposcopy | Dutch guideline^4^ | Perform see-and-treat approach for patients with high-grade colposcopic impression | Guideline adherence |
| Management strategy | 1. Treatment CIN < 1 | LEEP within 3 months after biopsy CIN < 1 | Biopsy result CIN < 1 after colposcopy | Dutch guideline^4^ | Do not treat biopsy CIN 1 | Overtreatment |
| Management strategy | 1. Treatment CIN 2^2^ | LEEP within 3 months after biopsy CIN 2 | Biopsy result CIN 2 after colposcopy | Dutch guideline^4^ | Shared decision making whether or not to treat biopsy CIN 2 | Overtreatment (for patients in fertile stage of life) |
| Management strategy | 1. Treatment CIN 3 | LEEP within 3 months after biopsy CIN 3 | Biopsy result CIN 3 after colposcopy | Dutch guideline^4^ | Perform treatment for patients with biopsy CIN 3 | Guideline adherence |
| Outcome of treatment | 1. Treatment specimen CIN | Treatment specimen  6.1 CIN < 1 after LEEP  6.2 CIN 2 after LEEP  6.3 CIN > 3 after LEEP | Total of LEEPs performed | Dutch guideline^4^ | 6.1 Do not treat  6.2 Shared decision making  6.3 Perform treatment | 6.1 Overtreatment  6.2 Overtreatment (for patients in fertile stage of life)  6.3 Guideline adherence |
| Outcome of treatment | 1. Normalization rate CIN 2 | No. of patients with < Pap 2 cytology at follow-up after LEEP | No. of patients with treatment specimen CIN 2 after LEEP^2^ | National Health Care Institute^18^ | - | Treatment success |
| Outcome of treatment | 1. Normalization rate CIN 3 | No. of patients with < Pap 2 cytology at follow-up after LEEP | No. of patients with treatment specimen CIN 3 after LEEP^2^ | National Health Care Institute^18^ | - | Treatment success |
| Follow-up | 1. Timeframe follow-up cytology after wait-and-see colposcopy with biopsy CIN 1 or low-grade referral cytology without biopsy at colposcopy   9.1 Between 10-14 months  9.2 < 10 months  9.3 > 14 months | No. of patients for whom follow-up cytology (with or without HPV) was performed | No. of patients with colposcopy (without treatment) and biopsy CIN 1 or low-grade referral cytology without biopsy at colposcopy | Dutch guideline^4^ | Perform follow-up cervical cytology at 12 months | Guideline adherence |
| Follow-up | 1. Timeframe follow-up cytology after LEEP with treatment specimen CIN 2    1. Between 4-8 months    2. < 4 months    3. > 8 months | No. of patients who had follow-up cytology performed (with or without HPV) | No. of patients who were treated with LEEP and treatment specimen showed CIN 2 | Dutch guideline^4^ | Perform follow-up cervical cytology at 6 months | Guideline adherence |
| Follow-up | 1. Timeframe follow-up cytology after wait-and-see colposcopy with biopsy CIN 2    1. Between 10-14 months    2. < 10 months    3. > 14 months | No. of patients who had follow-up cytology performed (with or without HPV) | No. of patients who underwent colposcopy (without treatment) and biopsy CIN 2 | Dutch guideline^4^ | Perform follow-up cervical cytology at 12 months | Guideline adherence |
| Follow-up | 1. Timeframe follow-up cytology after LEEP with treatment specimen CIN 3   12.1 Between 4-8 months  12.2 < 4 months  12.3 > 8 months | No. of patients who had follow-up cytology performed (with or without HPV) | No. of patients who were treated with LEEP and treatment specimen showed CIN 3 | Dutch guideline^4^ | Perform follow-up cervical cytology at 6 months | Guideline adherence |

1. For women not in fertile stage of life and the colposcopy shows a high-grade impression, a see-and-treat approach could be considered. [↑](#footnote-ref-1)
2. Also stratified by age category (< 40 years and > 40 years), as proxy for having completed childbearing. [↑](#footnote-ref-2)
