# Supplementary material for: Identification of over- and undertreatment in the Dutch national cervical cancer screening program: A data linkage study at the hospital level
Source: Prev Med Rep. 2023 Feb 10;32:102134. doi: 10.1016/j.pmedr.2023.102134 (PMC9958351; doi:10.1016/j.pmedr.2023.102134)
Supplement: Supplementary Table A.2 [file mmc6.docx]

**Table A.2: Histological outcome stratified by biopsy result**

| Biopsy | Treatment Specimen | | |
| --- | --- | --- | --- |
| CIN 1 (N=749) | CIN 1 or less: | 384 | 51% |
|  | CIN 2: | 199 | 27% |
|  | CIN 3 or more: | 166 | 22% |
| CIN 2 (N=3.682) | CIN 1 or less: | 950 | 26% |
|  | CIN 2: | 1822 | 49% |
|  | CIN 3 or more: | 910 | 25% |
| CIN 3 (N=5.030) | CIN 1 or less: | 565 | 11% |
|  | CIN 2: | 807 | 16% |
|  | CIN 3 or more: | 3658 | 73% |
